# The efficacy and safety of metoclopramide in relieving acute migraine attacks compared with other anti-migraine drugs: a systematic review and network meta-analysis of randomized controlled trials

**DOI:** 10.1186/s12883-023-03259-7

**Published:** 2023-06-08

**Authors:** Hanaa Abdelmonem, Hebatallah Mohamed Abdelhay, Gehad Taha Abdelwadoud, Amira Naser Mohammed Alhosini, Ahmed Eissa Ahmed, Samaher Walied Mohamed, Nada Mostafa Al-dardery, Mohamed Abd-ElGawad, Mohamed Abdelmonem Kamel

**Affiliations:** 1 MBBCh, Faculty of Medicine, Fayoum University, Fayoum, Egypt; 2 Clinical Oncology Resident at Fayoum University Hospital, Fayoum, Egypt; 3 Faculty of Medicine, Fayoum University, Fayoum, Egypt; 4 Family Medicine Resident at Fayoum University Hospital, Fayoum, Egypt

**Keywords:** Migraine, Metoclopramide, Nausea, Vomiting

## Abstract

**Background:**

Many drugs are prescribed in relieving acute migraine attacks, we aim to compare metoclopramide with other antimigraine drugs.

**Methods:**

We searched online databases like PubMed, Cochrane Library, Scopus, and Web of Science till June 2022 for RCTs that compared metoclopramide alone with placebo or active drugs. The main outcomes were the mean change in headache score and complete headache relief. The secondary outcomes were the rescue medications need, side effects, nausea and recurrence rate. We qualitatively reviewed the outcomes. Then, we performed the network meta-analyses (NMAs) when it was possible. which were done by the Frequentist method using the MetaInsight online software.

**Results:**

Sixteen studies were included with a total of 1934 patients: 826 received metoclopramide, 302 received placebo, and 806 received other active drugs. Metoclopramide was effective in reducing headache outcomes even for 24 h. The intravenous route was the most chosen route in the included studies and showed significant positive results regarding headache outcomes; however, the best route whether intramuscular, intravenous, or suppository was not compared in the previous studies. Also, both 10 and 20 mg doses of metoclopramide were effective in improving headache outcomes; however, there was no direct comparison between both doses and the 10 mg dose was the most frequently used dosage.

In NMA of headache change after 30 min or 1 h, metoclopramide effect came after granisetron, ketorolac, chlorpromazine, and Dexketoprofen trometamol. Only granisetron’s effect was significantly higher than metoclopramide’s effect which was only significantly higher than placebo and sumatriptan. In headache-free symptoms, only prochlorperazine was non-significantly higher than metoclopramide which was higher than other medications and showed significantly higher effects only with placebo. In rescue medication, metoclopramide’s effect was only non-significantly lower than prochlorperazine and chlorpromazine while its effect was higher than other drugs and showed higher significant effects only than placebo and valproate. In the recurrence rate, studies showed no significant difference between metoclopramide and other drugs. Metoclopramide significantly decreased nausea more than the placebo. Regarding side effects, metoclopramide showed a lower incidence of mild side effects than pethidine and chlorpromazine and showed a higher incidence of mild side effects than placebo, dexamethasone, and ketorolac. The reported extrapyramidal symptoms with metoclopramide were dystonia or akathisia.

**Conclusion:**

A dose of 10 mg IV Metoclopramide was effective in relieving migraine attacks with minimal side effects. Compared to other active drugs, it only showed a lower significant effect compared with granisetron regarding headache change while it showed significantly higher effects only with placebo in both rescue medication needs and headache-free symptoms and valproate in only rescue medication need. Also, it significantly decreased headache scores more than placebo and sumatriptan. However, more studies are needed to support our results.

**Supplementary Information:**

The online version contains supplementary material available at 10.1186/s12883-023-03259-7.

## Introduction

Migraine is a complex disorder of a neurovascular nature characterized by a severe throbbing pulsatile pain striking one side of the brain or being localized in the cerebral cortex or the brain stem frequently is accompanied by nausea, vomiting, and extreme sensitivity to light, sound, and smell with a high tendency of recurrence [1]. Moreover, migraine can be preceded by multiple sensory disturbances such as flashes of light or blind spots, or other disturbances such as tingling or numbness on one side of the face, arm, or leg [2]. Migraine is the world’s 7th leading cause of disability [3]. Every year, over a million adult patients attend US Emergency Departments to receive medical care for acute migraine attacks [4, 5].

The high prevalence of migraine and the severity of symptoms make the essence of management of the disease lie in finding a pain reliever with the least adverse effects and the most preventive of relapses. Various drugs have been used as metoclopramide a non-phenothiazine dopamine antagonist [6, 7], non-steroidal anti-inflammatory drugs such as ketorolac [8], a phenothiazine dopamine agonist such as prochlorperazine and chlorpromazine [9, 10], antiepileptic medication as valproate [11, 12], granisetron a potent selective antagonist of 5-HT3 receptor [13], corticosteroids such as dexamethasone [14] and serotonin agonists like sumatriptan [15].

Metoclopramide – a central dopamine receptor blocker [7] with peripheral muscarinic agonistic action [6] and anti-emetic effects [16] – is widely regarded as an effective choice for alleviating pain and nausea, therefore, considered an effective single agent for the treatment of migraine in ED [17]. Previous studies have mostly supported the use of this medication [18–20] others were not compatible with this [15, 21, 22] indicating a conflict about its efficacy. The objective of the study is to assess metoclopramide; its efficacy, side effects, and recurrence compared to other described migraine drugs in the literature.

## Methods

This review follows the updated Preferred Reporting Items for Systematic Reviews and Meta-Analyses (PRISMA) statement [23].

### Search strategy

We performed a broad search using four electronic databases like Scopus, PubMed, Web of Science, and Cochrane Library using the following search strategy from the inception till June 2022: (((((((((((((Metoclopramide) OR Metoclopramide[MeSH Terms]) OR Primperan) OR Reglan) OR Cerucal) OR Metoclopramide Dihydrochloride) OR Metoclopramide Hydrochloride) OR Rimetin) OR Maxolon) OR Metaclopramide) OR Metoclopramidum)) AND (((((((((((Migraine Disorders) OR Migraine Disorders[MeSH Terms]) OR Migraines) OR Migraine Headache) OR Acute Confusional Migraine) OR Status Migrainosus) OR Hemicrania Migraine) OR Migraine Variant) OR Headache) OR Abdominal Migraine) OR Cervical Migraine Syndrome).

### Eligibility criteria

We included all randomized clinical trials that investigated the effect of metoclopramide alone without any combination with an active drug in relieving acute migraine attacks whether it was compared with a placebo or any other active anti-migraine drugs like prochlorperazine, chlorpromazine, ketorolac, valproate, sumatriptan, bupivacaine, granisetron, dexketoprofen trometamol, dexamethasone, magnesium sulfate, pethidine, sumatriptan, and ibuprofen. Also, any dose or route of administration of metoclopramide was included. The primary outcomes were headache change and complete headache relief while the secondary outcomes were the recurrence of attacks, use of rescue drugs, nausea relief, and side effects. We excluded studies that combined metoclopramide with any other active drug, reviews, observational studies, case reports, case series, conference abstracts, and published articles in any language rather than English.

### Screening and study selection

The literature search results were exported to EndNote X8.0.1 and then to Excel software to start screening. We first independently screened the title and abstracts of each record. Then, the full texts of the remained articles were screened according to the eligibility criteria. Any conflict about the final decision of inclusion of any article was managed by discussion.

### Data extraction

The authors extracted the required data in the form of extraction Excel sheets. The extraction sheets included the following: (1) the summary of the included studies such as study design, inclusion and exclusion criteria, study groups, used headache scale, migraine medication use, the conclusion of the study, and time points of the assessed outcomes; (2) the baseline characteristics of the population of each study like age, gender, migraine headache type, attacks per year, onset/duration of the attacks, and migraine with aura incidence; (3) the outcomes of the studies which were headache change defined by the difference between headache score after a certain point of follow-up and the baseline score, headache relief defined by the complete absence of headache symptom, using of another rescue medication for relieving of the attack, recurrence of migraine attacks, side effects, nausea score change from certain follow-up point and the baseline, and nausea or emesis relief; and (4) risk of bias domains. All the authors extracted the data independently and the disagreements were solved by discussion among the members.

### Quality assessment

We follow the Risk of Bias tool 1 presented in the Cochrane Handbook for Systematic Reviews of Interventions in the assessment of quality [24]. We concluded the following domains in the assessment: sequence generation (selection bias), allocation sequence concealment (selection bias), blinding of participants and personnel (performance bias), blinding of outcome assessment (detection bias), incomplete outcome data (attrition bias), selective outcome reporting (reporting bias), and other potential sources of bias. Then, we categorized the judgments as low, high, or unclear.

### Statistical analysis and qualitative reporting

At first, we qualitatively reviewed each outcome as there were many different comparators and a limited number of studies was published for each comparator. Also, the outcomes were reported at different time points. All of these factors made performing direct meta-analyses difficult as it will include a small number of patients and this may lead to wrong interpretation of the results which made the priority to present a systematic review of the efficacy and safety of metoclopramide compared with other comparators in all time points as presented in the included studies.

Then, to support this systematic review with a direct meta-analysis when possible using the Review Manager 5.3 software.

Finally, we managed to perform network meta-analyses (NMAs) for the major outcomes like headache change, headache relief, and rescue medication need to compare the effect of metoclopramide 10 mg to other drugs. We chose specific time points of these outcomes that mostly were presented in the included studies which were headache change in periods ranged from 30 min to 1 h, complete headache relief in periods ranged from 45 min to 2 h, and rescue medication need in periods ranged from 30 min to 1 h. The NMAs were performed using Frequentist analysis by MetaInsight online software that used R-shiny and netmeta [25]. For all NMAs, three presented graphs were obtained: A) the network plot in which the nodes represented the number of patients in each group and the lines represented the number of direct comparisons between drugs, B) the forest plot showed the effect estimate of each drug compared to the reference group (metoclopramide), and C) the league table that arranged the drugs according to their superiority.

The headache change was pooled using the Standardized Mean Difference (SMD) with a 95% Confidence Interval (CI) as the included studies used different scales in outcome assessment like Visual Analogue Scale (VAS) and Numeric Rating Scale (NOS). While the categorical outcomes were analyzed using the Odds Ratios (ORs) with 95%CI. The outcomes were considered to be significant when the *P* values were < 0.05. The heterogeneity was assessed by the I^2^ test. Significant heterogeneity was considered if the I^2^ ≥ 50% or the *P* value became <0.1 [26]. The fixed effect model was used as a default for the analysis of homogeneous outcomes. In the case of heterogeneity, the random effect model was used. Then, a sensitivity analysis was performed to get the study that mostly affected the heterogeneity and the results were presented before and after the exclusion of this study.

## Results

### Literature search result

**Fig. 1:**
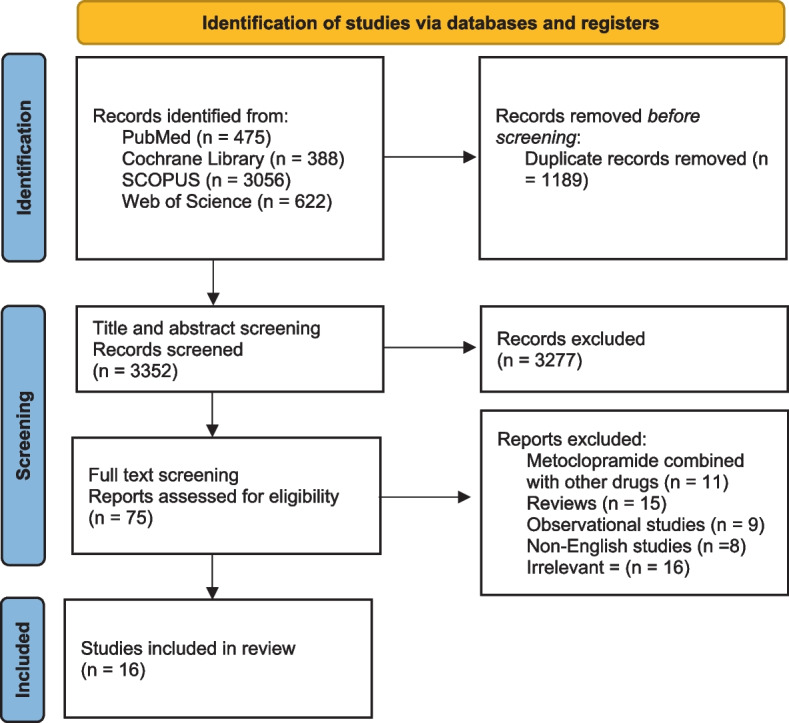
PRISMA flow diagram. *From:* Page MJ, McKenzie JE, Bossuyt PM, Boutron I, Hoffmann TC, Mulrow CD, et al. The PRISMA 2020 statement: an updated guideline for reporting systematic reviews. BMJ 2021;372:n71. doi: 10.1136/bmj.n71

Our literature search resulted in 3352 non-duplicated studies. We screened the titles & abstracts followed by the full texts of the 75 studies to check their eligibility. Finally, there were 16 randomized clinical trials included in our meta-analysis [15, 18, 19, 22, 27–38]. PRISMA flow diagram presented the full details of search results and study selection, Fig. 1.

### General and baseline characteristics

We included sixteen randomized clinical trials [15, 18, 19, 22, 27–38]. From them, we reviewed the data of 1934 patients; 826 of them received metoclopramide, while the rest of the patients received either a placebo (302) or different drugs (806) as a control group. Eight studies compared the effect of metoclopramide against placebo [15, 18, 19, 22, 27, 34–36]. However, thirteen studies compared metoclopramide with different drugs like prochlorperazine [15, 27], chlorpromazine [28, 33], ketorolac [28, 38], sumatriptan [29, 30], ibuprofen [36], magnesium sulfate [19], pethidine [34], valproate [38], granisetron [32], dexamethasone [28], dexketoprofen trometamol [31], and bupivacaine [37].

On the other hand, fourteen studies used the dose of 10 mg Intravenous (IV) of metoclopramide [15, 18, 19, 28, 29, 31, 32, 34–38], while two studies used 10 mg Intramuscular (IM) metoclopramide [22, 27]. Also, one study used the dose of 20 mg IV metoclopramide [30], and another one used the same dose (20 mg) IM instead of IV [22]. Finally, one study adjusted the dose to 0.1 mg/kg IV of metoclopramide [33], (Tables 1 and 2).

**Table 1:** General characteristics of the included studies

**ID**	**Country**	**Study design**	**Inclusion criteria**	**Exclusion Criteria**	**Migraine attack**	**Study groups**	**Time report**	**Scale of pain**	**Use of migraine medication**	**Diagnosis of migraine**	**Conclusion**
Friedman et al. 2020 [37]	US	Double-dummy RCT	• Age ≥18 years • Presentation to the ED with moderate / severe H/A that met International Classification of Headache Disorders, 3rd edition migraine criteria - with or without aura - probable migraine with aura - probable migraine without aura, provided they had ≥1 similar attack previously - Status migrainosus, prolonged duration of H/A (>72 h) - Early presentation (< 4 h)	Having at least one of the following: • Consent could not be obtained • Secondary cause of headache • Contraindications to GONB (skull defect, suspected infection overlying injection site, known bleeding disorder, ongoing use of antiplatelet agents or anticoagulants) • prior treatment with GONB • Contraindication or allergy to the study drugs • Pregnant / lactating	Acute attack	Metoclopramide 10 mg IV drip + sham GONB consisting of 6 ml saline (48 patients) Bupivacaine 0.5% 6 ml + IV drip normal saline (51 patients)	Baseline 1 h	11-point scale 0 = no pain, 10 = imaginable pain & Descriptive headache intensity scale none, mild, moderate, severe	Metoclopramide group: 36 patients received drugs for headache before ED visit Bupivacaine group: 32 patients received drugs for headache before ED visit	International Classification of Headache Disorders, 3rd edition migraine criteria 2013	Authors conclude that metoclopramide could improve pain intensity more than GONB as first line for ttt acute migraine attack
						Both drugs injected adjacent to greater occipital nerve bilaterally, Administrated over 15 min					
Yavuz et al. 2020 [31]	Turkey	Superiority trial prospective double blinded RCT	• Age: ≥ 18 • Patients were diagnosed as acute migraine headache by emergency medicine physician according to diagnostic criteria of the International Headache Association • Patients were enrolled in the study consecutively 24 h a day, 7 days a week	• Refused to participate in the study • Pregnancy • Taking any analgesic medication within 6 h before presentation to ED • Having other diseases (e.g., hypertension, hypoglycemia, chronic kidney failure, and intracranial masses) • Hemodynamically unstable • Allergy to study drugs	Acute attack	Metoclopramide 10 mg IV (50 patients) Dexketoprofen trometamol 50 mg IV (50 patients) Metoclopramide 10 mg IV + Dexketoprofen 50 mg (50 patients)	Baseline 15 min 30 min	VAS	--	International Headache Association 2004	At 15 min, there was non-significant difference between gps (*p* = 0.09) At 30 min: the combination could significantly decrease pain more than other gps (*p* = 0.004)
Khazaei et al. 2019 [28]	Iran	prospective double blinded RCT	• Headaches which met the IHS criteria for migraine • Episodic migraine and did not take preventive therapies • Patients did not have repeated referrals to ED • Headache intensity was higher than 4 on VAS	Having at least one of: • Hypertension • Renal or hepatic failure • Cardiac or respiratory disease • Malignancy • Acute inflammatory disease or infection • Peptic ulcer • Pregnancy / lactation • Neurological deficit • History of immunesuppressive drugs • Epilepsy • Ergotamine use in the past 8 h • Anxiolytic use in the previous 4 h • Life threatening causes of H/A: SAH, meningitis, arterial dissection, or high intracranial pressure	Acute attack	Metoclopramide 10 mg IV (32 patients) Dexamethasone 8 mg IV (32 patients) Chlorpromazine 25 mg IV (32 patients) Ketorolac 30 mg IV (32 patients)	Baseline 1 h 24 h	VAS	All patients didn’t take preventive therapies	International Headache Society (HIS) 2004	Authors conclude that all drugs could significantly decrease pain of acute migraine attack (*p* < 0.05); however, there was non-significant difference between the four drugs (*p* = 0.07)
Doğan et al. 2019 [35]	Turkey	Prospective double blinded RCT	• Age: >18 years old • Presentation to the ED with migraine‐type headaches and met International Headache Society (IHS) migraine criteria	• Taking analgesic drugs during the previous 2 h • Pregnant • Had documented or declared allergies to metoclopramide • Hemodynamically unstable • Refuse to participate in this research	Acute attack	Metoclopramide 10 mg (74 patients) Normal saline 100 ml (74 patients)	Baseline 15 min 30 min	NRS	metoclopramide group: 69 pts had history of migraine, 18 pts use migraine drugs Placebo group: 65 pts had history of migraine, 24 pts use migraine drugs	International Headache Society (IHS) migraine criteria 3rd edition beta version	Authors conclude that there was no difference between metoclopramide and saline for ttt acute attack of migraine
						Both drugs infused over 10 min					
Amiri et al. 2017 [32]	Iran	Prospective double blinded RCT	• Age: >18 years old • Presenting with headache with a previous history of migraine headache diagnosed by a neurologist	• Pregnant / lactating • Suddenly initiated headache (different from the previous attacks) • Abnormal neurologic findings or head trauma within the last month • Uncooperative • Needed for additional doses of morphine • Had an uncertain diagnosis	Acute attack	Metoclopramide 10 mg IV bolus (73 patients) Granisetrone 2 mg IV bolus (75 patients)	Baseline 1 h 2 h 4 h	VAS	--	Diagnosis by neurologist	Authors conclude that grabisetrone could significantly decrease pain more than metoclopramide (*p* = 0.03)
Friedman et al. 2014 [38]	US	Double blinded RCT	• Acute migraine or acute probable migraine headache as defined by the International Headache Society’s International Classification of Headache Disorders, second edition 2004 • Patients with acute probable migraine were only eligible if their reason for not meeting full migraine criteria was insufficient number of total lifetime headaches or prolonged duration of headache (72 h)	• Secondary headache • Receiving a lumbar puncture in the ED • Temperature ≥100.4°F • Having a new neurologic abnormality, seizure disorder • Concurrent use of study drugs / monoamine oxidase inhibitor / immunesuppressives • Pregnancy, lactation • Previous enrollment • Allergy • Intolerance • Contraindication to the study drugs, including hepatic dysfunction, peptic ulcer	Acute attack	Metoclopramide 10 mg IV (110 patients) Ketorolac 30 mg IV (110 patients) Valproate 1 g IV (110 patients)	Baseline 1 h	NRS & Descriptive ordinal scale	Two in metoclopramide gp Two in ketorolac gp Three in valproate gp All of them took preventive migraine medication	International Headache Society’s International Classification of Headache Disorders, second edition 2004	Authors couldn’t identify a clear vision; however, they thought that valproate was the least effective while metoclopramide was better than ketorolac in decrease pain of acute migraine attack
						All drugs were IV over 15 min					
Talabi et al. 2013 [30]	Iran	Double blinded RCT	• Age: 20–60 years old • Presented with acute headache similar to previous episodes with or without phonophobia, photophobia, vomiting, or nausea	• Fever • Neck stiffness • Altered mental state • Pregnancy • Hypertension • Recent trauma or seizure (within 24 h) • Focal neurological abnormality on physical examination • Allergy to metoclopramide • Cardiovascular diseases • Taking triptan or ergot during the last 24 h	Acute attack	Metoclopramide 20 mg IV Infused over 15 min (62 patients) Sumatriptan 6 mg SC (62 patients)	Baseline 1 h	VAS	--	International Headache Society (IHS)	Authors conclude that metoclopramide could significantly decrease pain intensity of acute attack of migraine (*p* < 0.001)
Salazar-Zúñiga et al. 2006 [29]	México	RCT	• Age: 18 to 65, of either sex • Moderate to severe intensity • With or without aura • Without restriction in time of evolution of the migraine • History of one to six attacks in 1 month	• Fever prior to pain • Use of psychotropic drugs, tobacco or alcohol in the last 24 h • Coronary disease • Recent trauma, meningism • Three months previous use of drugs: (barbiturate, antihypertensive, antiparkinsonian, antihistamine, anticholinergic) • Morbid obesity	Acute attack	Metoclopramide 10 mg IV Slowly infusion over 3–5 min (60 patients) Sumatriptan 6 mg SC Slowly (60 patients)	Baseline 15 min 30 min 45 min 1 h	Headache intensity scale; 0 = no headache 1 = mild 2 = moderate 3 = intense	--	International Headache Society criteria for migraine 2004	Authors conclude that metoclopramide could significantly decrease pain more than sumatriptan only at 15 min (*p* < 0.01)
Cicek et al. 2004 [34]	Turkey	Prospective double blinded RCT	Having ≥4 symptoms of vascular score criteria: • Aura or anticipation of H/A, nausea, vomiting, diarrhea, anorexia • Unilateral / Periodical / Throbbing headache • Photophobia / phonophobia • Visual complaints • Childhood onset H/A • History of motion sickness • FH of headache • Headache triggered by certain foods • Temporal association with menstruation	• Secondary headache • Altered mental status • Abnormal vital signs • Pregnant • History of epilepsy • Parkinson • Pheochromacytoma • Known allergy to the study drugs	Acute attack	Metoclopramide 10 mg IV + Placebo IM (85 patients) Placebo IV + Placebo IM (83 patients) Pethidine 50 mg IM + Placebo IV (84 patients) Metoclopramide 10 mg IV + Pethidine 50 mg IM (84 patients)	Baseline 15 min 30 min 45 min	VAS	--	Vascular score criteria	Metoclopramide had the same effect as the combination (*p* = 1) Also, metoclopramide alone or the combination could significantly decrease pain more than pethidine alone (*p* = 0.038)
Cete et al. 2005 [19]	Turkey	Prospective double blinded RCT	• Nonconsecutive sample of patients who presented to the ED with headache, meeting International Headache Society criteria for migraine	• Age <18 years old • A known adverse reaction to metoclopramide or MgSO4 • Consumption of the study drugs within 48 h • Temperature of ≥ 38 • Altered mental status, meningeal signs • Renal or cardiac disease	Acute attack	Metoclopramide 10 mg IV + normal saline 100 ml (37 patients) Normal saline 100 ml (40 patients) MgSO4 2 g + normal saline 100 ml (36 patients)	Baseline 15 min 30 min	VAS	--	International Headache Society criteria 1988	At both 15 and 30 min: All drugs could significantly decrease pain (*p* < 0.001); however, the difference between them was non-significant (0.619)
						All drugs infused over 10 min					
Jones et al. 1996 [21]	US	Prospective double blinded RCT	• Age: ≥ 16 years old • Had normal ability to communicate • Had one or more of the following: - Recurrent headaches preceded by neurological symptoms - Recurrent throbbing headaches that were at least initially unilateral - Recurrent throbbing headaches consistently associated with significant nausea or vomiting, photophobia, sonophobia, or mood changes	• Age >60 years • A known intolerance to study drugs • Using drugs that cause extrapyramidal reactions • Pregnancy or lactation • History of drug-seeking behavior • Lack of responsible person available to care for and transport the patient when departing from the ED • Secondary causes of H/A e.g. neurological or seizure disorder, toxic exposure, alcohol abuse, vascular disease, recent trauma, hypertension, or hepatic or renal failure	Acute attack	Metoclopramide 10 mg IM (29 patients) Normal saline 2 ml IM (29 patients) Prochlorperazine 10 mg IM (28 patients)	Baseline 1 h	VAS	many pts use drugs before presentation into ED	International Headache Society classification 1988	Authors conclude that prochlorperazine could significantly decrease pain more than metoclopramide (*p* < 0.01)
Coppola et al. 1995 [15]	Texas	Prospective double blinded RCT	• Age 18–65 years old • Presentation to the ED with migraine headache as defined by the Ad Hoc Committee on Classification of Headache • Patients were enrolled if they presented with cephalgia similar to previous episodes, with or without nausea, vomiting, photophobia, or phonophobia	• Fever or meningismus • Altered mental state • Recent (within 24 h) use of analgesics, drugs, or alcohol • pregnancy • Oxygen saturation less than 90% • Recent trauma or seizure (within 24 h) • First episode of headache • Suspicion of intracranial process requiring further investigation (eg, SAH, space-occupying lesion) • Allergy to study drugs • Diastolic BP >90 mm Hg	Acute attack	Metoclopramide 10 mg IV (24 patients) Normal saline 2 ml IV (24 patients) Prochlorperazine 10 mg IV (22 patients)	Baseline 30 min	VAS	--	Ad Hoc Committee on Classification of Headache 1962	Authors conclude that there was non-significant difference between drugs in decreasing pain of acute migraine attack (*p* = 0.15)
Cameron et al. 1995 [33]	London	Prospective double blinded RCT	• Aged 17–60 years old • Presenting to hospital ED, diagnosed as having acute migraine headache by the attending physician • Diagnosis of migraine either with or without aura, as defined by the International Headache society 1988 • Patients taking opioids were not excluded	• First migraine or nonmigraine headache • More than six prior headaches per month • Allergy to study drugs • Present use of phenothiazine, isoniazid, monoamine oxidase (MAO) inhibitor, or cyclic antidepressant • Parkinson’s disease or a past history of dystonic reactions • Seizure disorder • Age <17 or >60 years • Pregnancy or lactation • Lack of transportation home / no person to care for the patient at home • Lack of consent	Acute attack	Metoclopramide 0.1 mg/kg IV (44 patients) Chlorpromazine 0.1 mg/kg IV (47 patients) Both drugs diluted to a concentration of 2.5 mg/ml	Baseline 45 min	VAS	No prior use of ttt Or Taking acetaminophen/ aspirin / NSAID failure	criteria of International Headache society 1988	Authors conclude that there was non-significant difference between drugs in decreasing pain of acute migraine attack (*p* = 0.35)
									(10/34) Pts taking codeine preparations / oxycodone and (5/16) pts taking butalbital preparation failed to respond to ttt		
Ellis et al. 1993 [36]	US	double blinded RCT	• Age: ≥ 18 years old • Normal ability to communicate • Had one or more of the following characteristics: - recurrent H/A that were at least initially unilateral /proceeded by neurologic symptoms - recurrent throbbing H/A that consistently associated with significant nausea or vomiting, or associated with mood changes, and photophobia	• Taking monoamine-oxidase inhibitors • History of pheochromocytoma or epilepsy • Receiving other drugs likely to cause extrapyramidal reactions • Pregnant and breast-feeding	Acute attack	Metoclopramide 10 mg IV + Placebo oral (10 patients) Placebo oral + Placebo IV (10 patients) Ibuprofen 600 mg oral + Placebo IV (10 patients) Metoclopramide 10 mg IV + Ibuprofen 600 mg oral (10 patients)	Baseline 30 min 1 h	VAS	--	--	Metoclopramide could significantly decrease pain more than ibuprofen at both 30 min and 1 h. (*p* = 0.04, *p* = 0.01 respectively) However, the difference between metoclopramide and the combination wasn’t significant
Tek et al. 1990 [18]	California	Prospective double blinded RCT	• Diagnosis of migraine was made by physicien if the patient had a periodic headache with a throbbing component • Presence of at ≥1 of the following symptoms: photosonophobia, nausea or vomiting, unilaterality, strong FH, onset in adolescence, or history of relief with ergot • The history of aura combined with the above criteria differentiated the headache as a classic (VS common) migraine	• Age: <18 or >60 years • First episode of headache • Sudden onset of headache • History of recent trauma or seizure • Fever, meningismus, • Altered mental status • Focal neurological abnormality • Primary diagnosis other than migraine • Having any contraindication to the study drug	Acute attack	Metoclopramide 10 mg IV (24 patients) Normal saline 2 ml IV (26 patients)	Baseline 1 h	Relief score / numeric scale	--	Ad Hoc Committee on Classification of Headaches 1962	Authors conclude that metoclopramide could significantly obtain pain relief (*p* < 0.02)
Tfelt-Hansen et al. 1980 [22]	Denmark	Double blinded RCT	• The trial involved 150 patients with classical or common migraine as defined by the Ad Hoc Committee on Classification of Headache • They fulfilled the criteria suggested by Olesen • Patients presented themselves with a migraine attack at the acute migraine clinic in Copenhagen • Patients with marked nausea or vomiting were selected		Acute attack	Metoclopramide 10 mg IM + Placebo suppository (49 patients) Placebo IM + Placebo suppository (51 patients) Metoclopramide 20 mg suppository + Placebo IM (50 patients)	Baseline 1 h	Rating scale of headache	--	Ad Hoc Committee on Classification of Headache 1962	Authors conclude that metoclopramide couldn’t significantly decrease pain either IM or suppository (*p* = 0.06)
						All patients were given paracetamol (aceteminophen) 1 g and diazepam 5 mg orally					

*RCT* Randomized Controlled Trial, *US* United States, *GONB* Greater Occipital Nerve Block, *ED* Emergency Department, *VAS* Visual Analogue Scale, *NRS* Numeric Rating Scale, *IV* Intravenous, *IM* Intramuscular, *SAH* Sub Arachnoid Hemorrhge, *BP* Blood pressure, *NSAID* Non-Steroidal Anti Inflammatory Drugs, *PP*-value, *h* hours, *min* minutes, *ttt* treatment, *gp* group, *pts* patients, *H/A* Headache, *FH* Family History, *VS* Versus

**Table 2:** Baseline characteristics of the included population

**ID**	**Study groups**	**Number of participants**	**Age (Years), mean (SD)**	**Gender (male), Frequency**	**Type of migraine headache**	**Attacks per year** **Mean (SD)**	**Onset /Duration of attacks in h** **mean (SD)**	**Migraine with aura**
Friedman et al. 2020 [37]	Metoclopramide 10 mg IV	48	38 (11)	14	--	--	D: 99 (159)	27
	Bupivacaine 0.5% (6 mL)	51	39 (11)	7	--	--	D: 100 (137)	26
Yavuz et al. 2020 [31]	Metoclopramide 10 mg IV	50	38.1 (11.9)	12	--	Median, IQR: 12 (6)	O: 4.4 (3.6)	13
	Dexketoprofen trometamol 50 mg IV	50	35.1 (11.3)	15	--	Median, IQR: 12 (5)	O: 5.6 (7.3)	15
Khazaei et al. 2019 [28]	Metoclopramide 10 mg IV	32	Total; Migraine with aura: 37.81 (9.27) Migraine without aura: 36.56 (10.1)	--	--	--	--	7
	Dexamethasone 8 mg IV	32		--	--	--	--	3
	Chlorpromazine 25 mg IV	32		--	--	--	--	11
	Ketorolac 30 mg IV	32		--	--	--	--	6
Doğan et al. 2019 [35]	Metoclopramide 10 mg IV	74	33.67 (13.33)	24	--	14.33 (12.59)	O: 7 (8.15)	23
	Normal saline 100 ml	74	33 (13.33)	28	--	19 (17.04)	O: 5.67 (4.44)	16
Amiri et al. 2017 [32]	Metoclopramide 10 mg IV	73	Total; Mean: 33.5	Total; Males = 47	--	--	--	--
	Granisetrone 2 mg IV	75			--	--	--	--
Friedman et al. 2014 [38]	Metoclopramide 10 mg IV	110	34.67 (13.33)	18	--	--	D: 88 (106.67)	52
	Ketorolac 30 mg IV	110	34.33 (14.07)	17	--	--	D: 86 (102.22)	53
	Valproate 1 gm IV	110	33 (11.85)	19	--	--	D: 72 (71.11)	53
Talabi et al. 2013 [30]	Metoclopramide 20 mg IV	62	34.9 (9)	39	--	--	--	--
	Sumatriptan 6 mg SC	62	26.8 (4)	38	--	--	--	--
Salazar-Zúñiga et al. 2006 [29]	Metoclopramide 10 mg IV	60	Range from 18–65	Total; Males: 9	--	--	--	--
	Sumatriptan 6 mg SC	60			--	--	--	--
Cicek et al. 2004 [34]	Metoclopramide 10 mg IV + Placebo IM	50	Total; 38.8 (11.1)	Total; Males: 23	--	--	--	--
	Placebo IV + Placebo IM	48				--	--	--
	Pethidine 50 mg IM + Placebo IV	49				--	--	--
Cete et al. 2005 [19]	Metoclopramide 10 mg IV + 100 ml normal saline	37	40 (13)	4	--	--	--	5
	Normal saline 100 ml IV	40	10 (11)	5	--	--	--	8
	MgSO4 2 mg + 100 ml normal saline	36	40 (12)	9	--	--	--	7
Jones et al. 1996 [21]	Metoclopramide 10 mg IM	29	Total; 32.1 (2.1)	Total; Males: 21	--	--	--	--
	Normal saline 2 ml IM	29			--	--	--	--
	Prochlorperazine 10 mg IM	28			--	--	--	--
Coppola et al. 1995 [15]	Metoclopramide 10 mg IV	24	--	--	--	--	--	--
	Normal saline 2 ml IV	24	--	--	--	--	--	--
	Prochlorperazine 10 mg IV	22	--	--	--	--	--	--
Cameron et al. 1995 [33]	Metoclopramide 0.1 mg/kg IV	44	31.6 Range: 19–54	9	--	--	D: 47.2 Range: 1 – 456	8
	Chlorpromazine 0.1 mg/kg IV	47	32.6 (66.46) Range: 17–55	9	--	--	D: 38.9 Range:1.5 – 576	7
Ellis et al. 1993 [36]	Metoclopramide 10 mg IV + Placebo oral	10	At least 18 years old	--	--	--	--	--
	Placebo oral and IV	10		--	--	--	--	--
	Ibuprofen 600 mg oral + Placebo IV	10		--	--	--	--	--
Tek et al. 1990 [18]	Metoclopramide 10 mg IV	24	Range: 18–60 years old	--	7 had classic migraine 39 had common migraine 4 not differentiated	--	--	--
	Normal saline 2 ml	26		--		--	--	--
Tfelt-Hansen et al. 1980 [22]	Metoclopramide 10 mg IM + Placebo suppository	49	Total; 43.25 (14) Range: 18–74	Total; Males: 17	Classical and common migraine	Median: 1.5 per month	Total; Attacks <4 h = 18 (12%) Attacks 4–24 h = 95 (63%) Attacks ≥25 h = 37 (25%)	--
	Placebo IM + Placebo suppository	51						--
	Metoclopramide 20 mg suppository + Placebo IM	50						--

*IV* Intravenous, *IM* Intramuscular, *SC* Subcutaneous, *SD* Standard Deviation, *IQR* Inter Quartile Range, *h* hour, *D* Duration, *O* Onset

### Results of risk of bias assessments

**Fig. 2:**
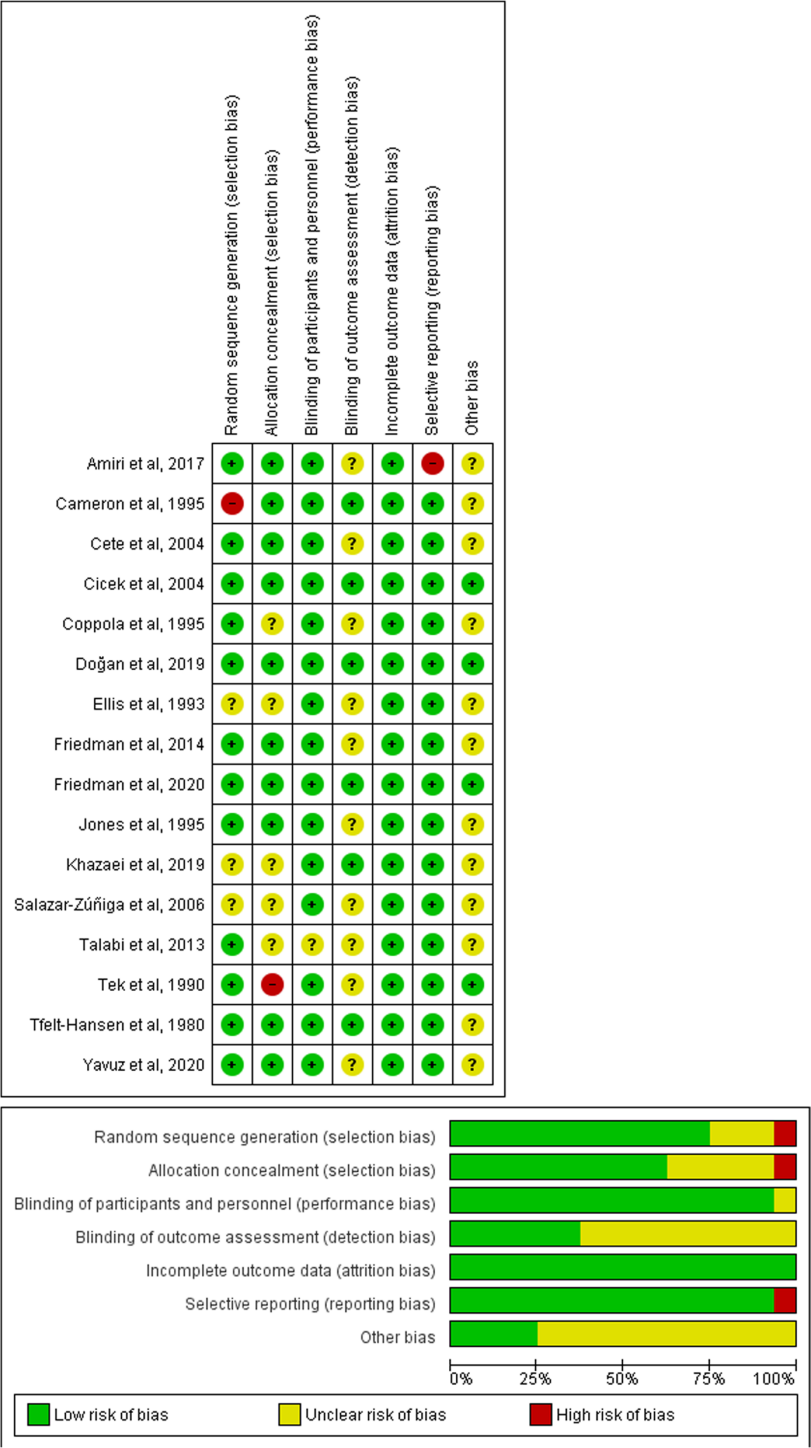
Risk of bias summary

According to Risk of Bias tool 1, quality assessment of the included studies revealed three articles with good quality [34, 35, 37], nine with fair quality [18, 19, 22, 28, 31–33, 36, 38], and four with poor quality [15, 29, 30, 36]. The details of each domain are presented in Fig. 2.

### Headache change (Supplementary Table 1)

Sixteen studies investigated the efficacy of metoclopramide on headache scores at different time points [15, 18, 19, 22, 27–38]. Ten studies used VAS in assessing headache scores [15, 19, 27, 28, 30–34, 36], two used NRS [35, 38], one used an 11-point scale with zero represented no pain and ten represented the highest pain [37], one used Headache Intensity Scale [29], one used Numeric Relief Scores [18], and one used Rating Scale of Headache [22] (Supplementary Table 1).

### Headache change in 15 min

Four studies measured headache scores after 15 min [19, 29, 31, 35]. The 10 mg of IV metoclopramide showed a more significant decrease in headache score than 6 mg Subcutaneous (SC) sumatriptan [29]. While it had a less significant decrease in headache score than 2 mg magnesium sulfate only in patients of migraine with aura [19] and non-significant effects compared to 50 mg IV dexketoprofen trometamol [31] and 100 ml IV normal saline [35].

### Headache change in 30 min

Six studies measured headache scores after 30 min [15, 19, 29, 31, 35, 36]. The 10 mg of IV metoclopramide showed more significant decreases in headache scores than placebo [36], 600 mg oral ibuprofen [36], and 10 mg IV prochlorperazine [15]. While it had a less significant decrease in headache score than placebo in one study [15]. Also, it showed non-significant differences in the other four studies compared to 2 mg magnesium sulfate [19], placebo [19, 35], 6 mg SC sumatriptan [29] and 50 mg IV dexketoprofen trometamol [31].

### Headache change in 45 min

Three studies measured headache scores after 45 min [29, 33, 34]. The 10 mg of IV metoclopramide showed more significant decreases in headache scores than placebo and 50 mg IM pethidine [34]. While it showed non-significant differences when compared to chlorpromazine [33] and sumatriptan [29].

### Headache change in 1 h

Ten studies measured headache scores at 1 h [18, 22, 27–30, 32, 36–38]. The 10 mg IV metoclopramide showed more significant decreases in headache scores than the placebo [36], and 600 mg ibuprofen [36]. However, it showed less significant decreases in headache scores than placebo [18], 2 mg IV granisetron [32] and 1 gm IV valproate [38]. Moreover, it showed non-significant differences compared to 6 mg SC sumatriptan [29], dexamethasone [28], chlorpromazine [28], ketorolac [28, 38], and 6 ml bupivacaine 0.5% [37].

On the other hand, the 10 mg IM metoclopramide showed a more significant decrease in headache score than saline while it showed a less significant decrease than 10 mg IM prochlorperazine [27]. While Tfelt-Hansen 1980 et al. showed that its effect did not significantly different from placebo or metoclopramide 20 mg suppository [22].

Also, the 20 mg IV metoclopramide showed a more significant decrease in headache score than 6 mg SC sumatriptan [30].

### Assessed headache change in durations of more than 1 h

Two studies measured headache scores after more than 1 h [28, 32]. The 10 mg of IV metoclopramide had less significant decreases in headache scores at both 2 and 4 h than 2 mg IV granisetron [32]. Moreover, it had shown non-significant differences at 24 h compared with dexamethasone [28], chlorpromazine [28] and ketorolac [28].

### Direct meta-analyses results of assessed headache change in durations between 15 min to 1 h

We succeeded only to perform the direct meta-analyses to compare metoclopramide to placebo and chlorpromazine. We found that the overall mean difference favored metoclopramide over placebo in pooled two studies [34, 35] (SMD = -0.63, 95% CI [-0.88 to -0.37], and a *P* value of <0.00001), pooled analysis was homogenous (*P* = 0.24). However, when compared with chlorpromazine in two studies [28, 33], the overall mean difference did not favor either of the two groups (SMD = 0.25, 95% CI [-0.07 to 0.56], and a *P* value of <0.13), pooled analysis was homogenous (*P* = 0.72), Supplementary Figs. 1 and 2.

### Headache relief and success of treatment

Ten studies assessed headache relief among patients at different time points [15, 18, 22, 27, 29, 33–35, 37, 38]. Three of them assessed it at intervals of more than 1 h [35, 37, 38], four at one [18, 22, 27, 29], three at 45 min [29, 33, 34], two at 30 min [15, 29], one at 15 min [29], (Supplementary Table 2).

At 15 min, the 10 mg IV of metoclopramide had a significantly higher incidence of headache relief than the 6 mg of SC sumatriptan [29].

At 30 min, the 10 mg of IV metoclopramide had a lower significant effect than the 10 mg IV prochlorperazine [15]. Also, it showed non-significant differences compared with placebo [15] and 6 mg of SC sumatriptan [29].

At 1 h, the 10 mg of IV metoclopramide had a higher significant effect than the placebo [18]; however, it showed a non-significant difference compared to the placebo in another study [29]. On the other hand, 10 mg IM metoclopramide had a lower significant incidence of headache relief than 10 mg IM prochlorperazine [27]. However, when compared with a placebo, it showed a higher significant incidence of headache relief in one study [27] and a non-significant difference in another study [22].

At 45 min, and intervals of more than 1 h, the 10 mg IV metoclopramide showed non-significant differences to sumatriptan in 45 min and placebo in 24 to 72 h of follow-up [29, 35].

We succeeded only to perform the direct meta-analysis to compare metoclopramide with placebo in durations between 15 min to 1 h [18, 27]. The OR favored metoclopramide over placebo which equalled 5.16, 95% CI [1.83 to 14.55], with a *P* value of 0.002. The pooled analysis was homogenous (*P* = 0.23), Supplementary Fig. 3.

### Rescue medication need

Ten studies assessed the need for additional analgesics [15, 19, 27, 31, 33–38]. Six of them had assessed the rescue of analgesics at 1 h [27, 33, 34, 36–38], while the other four at 30 min [15, 19, 31, 35]. They used different drugs as a rescue medication; four used drugs upon the choice of the treating physician [15, 34, 36, 38], two studies used parenteral narcotics like meperidine [19, 27], another two used 1 μg/kg of fentanyl [31, 35], and one study used the other study drug or meperidine with dimenhydrinate [33].

At 30 min, the 10 mg of IV metoclopramide had significantly lower incidences of the need for additional analgesics than normal saline or 2 mg of magnesium sulfate [19], while it had shown a non-significant difference compared with saline in another study [35].

At 1 h, the 10 mg of IV metoclopramide had significantly lower incidences of the need for additional analgesics than the placebo or 50 mg IM pethidine [34]. However, the 10 mg of IM metoclopramide had significantly higher incidences than the placebo or 10 mg IM prochlorperazine [27]. Also, there were non-significant differences between 0.1 mg/kg IV metoclopramide and 0.1 mg/kg IV chlorpromazine [33] besides 10 mg of IV metoclopramide and 6 ml bupivacaine 0.5% [37], (Supplementary Table 3).

We succeeded only to perform the direct meta-analysis to compare metoclopramide with placebo in durations between 30 min to 1 h which significantly favored the metoclopramide, OR = 0.28, 95%CI = 0.19—0.43, *P* < 0.00001 [15, 19, 27, 34–36]. Also, we managed to compare the metoclopramide to prochlorperazine and the analysis significantly favored prochlorperazine with OR = 3.02, 95%CI = 1.15 – 7.94, *P* = 0.03 [15, 27]. Both comparisons were homogeneous, *P* = 0.1 and *P* = 0.89 respectively, Supplementary Figs. 4 and 5.

### Recurrence of attacks

Six studies assessed the recurrence of migraine attacks in different intervals [15, 18, 19, 28, 33, 35]: two studies at 24 h [15, 19], three studies at 48 h [15, 18, 33], and one at 24–72 h [35]. At 48 h, two studies showed no recurrence [15, 18]. However, at the other intervals, there were non-significant differences between groups in each study, (Supplementary Table 4).

### Side effects

Extrapyramidal reactions in the form of dystonia or akathisia were reported by two studies for 10 mg of IV metoclopramide [15, 19], and 10 mg of IV prochlorperazine [15]. Also, it was treated with diphenhydramine. In addition, one study assessed akathisia by asking patients to rate their anxiety and restlessness on a 0–10 scale [37]. However, the 6 ml of bupivacaine 0.5% had a more significant protective effect than the 10 mg of IV metoclopramide regarding anxiety and a non-significant effect regarding restlessness [37].

Mild side effect as dizziness, drowsiness, gastrointestinal symptoms, anxiety, dry mouth and blurred vision were reported by eight studies [18, 27–29, 33–35, 38]. The 10 mg of IV Metoclopramide showed significantly lower incidences of mild side effects than 50 mg of IM pethidine [34] and 25 mg of IV chlorpromazine [28]. However, it showed significantly higher incidences of mild side effects than placebo [34], 8 mg of IV dexamethasone [28], and 30 mg of IV Ketorolac [28]. However, it showed no difference with normal saline in Dogan et al. [35]. On the other hand, the 0.1 mg/kg IV of metoclopramide showed a non-significant difference when compared with 0.1 mg/kg IV of chlorpromazine [33], (Supplementary Table 5).

### Nausea/emesis change

Four studies reported nausea/emesis changes [15, 22, 32, 36]. Three of them used VAS for the assessment of nausea/emesis scores [15, 32, 36], while the last one used the Rating scale of nausea [22].

Three studies reported nausea/emesis change at 1 h [22, 32, 36]. The 10 mg of IM metoclopramide or 20 mg of suppository metoclopramide showed a significant improvement in nausea/emesis in comparison to the placebo [22]. The 10 mg of IV metoclopramide showed a significant improvement in nausea/emesis scores when compared with 600 mg oral ibuprofen [36]. However, it showed a non-significant improvement when compared with 2 mg IV granisetron [32].

At 30 min, two studies reported nausea/emesis changes [15, 36]. Also, at more than 1 h, one study reported nausea/ emesis changes [32]. However, the 10 mg of IV metoclopramide showed non-significant differences at each time point of follow-up, (Supplementary Table 6).

### Nausea/emesis incidence

Three studies reported nausea/emesis incidences [27, 33, 34]. Only in one study, the 10 mg IM metoclopramide showed a significantly lower incidence than 2 ml IM normal saline and a higher incidence than 10 mg IM prochlorperazine [27], (Supplementary Table 7).

### The NMAs results

#### Headache change in durations between 15 min to 1 h

**Fig. 3:**
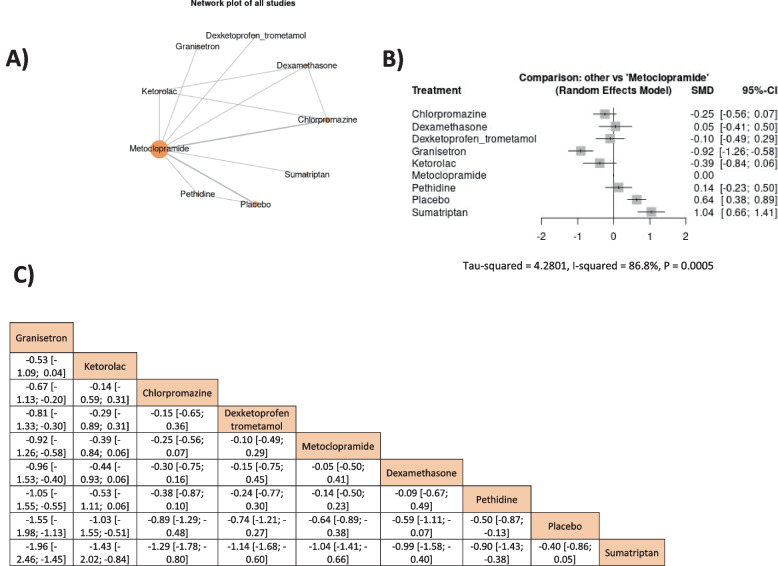
The network meta-analysis of headache changes in durations from 30 min to 1 h

A network meta-analysis of the results was presented in Fig. 3. The metoclopramide 10 mg came after granisetron, ketorolac, chlorpromazine, and dexketoprofen trometamol. Only granisetron’s effect was significantly higher than metoclopramide’s effect (SMD = -0.92, 95% CI = [-1.26, -0.58]); However, metoclopramide showed only a higher significant effect than placebo and sumatriptan, SMD = -0.64, 95% CI = (-0.89, -0.38) and SMD = -1.04, 95% CI = [-1.41, -0.66], respectively. The outcome was heterogeneous; therefore, a random effect model was used (I^2^ = 86.8%, *P* = 0.0005); however, the heterogeneity could not be solved by leaving a study out of the analysis, Fig. 3.

#### Complete headache relief in durations between 15 min to 1 h

**Fig. 4:**
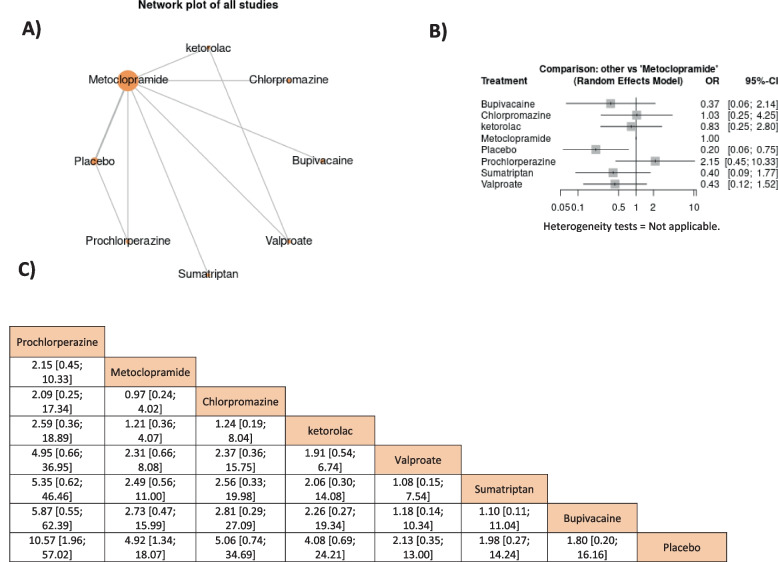
The network meta-analysis of headache-free symptoms in durations from 45 min to 2 h

Only prochlorperazine was non-significantly higher than metoclopramide 10 mg which was higher than other medications and showed significantly higher effects only with placebo, OR = 4.92, 95% CI = [1.34, 18.07], respectively, Fig. 4.

#### The rescue medication need in durations between 30 min to 1 h

**Fig. 5:**
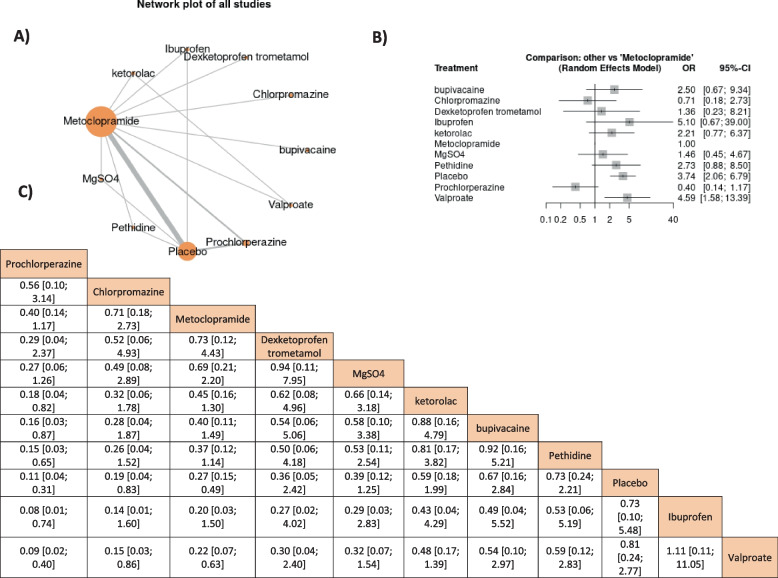
The network meta-analysis of rescue medication in durations from 30 min to 1 h

Metoclopramide’s effect was only non-significantly lower than prochlorperazine and chlorpromazine while its effect was higher than other drugs and showed higher significant effects only to placebo and valproate, OR = 0.27, 95% CI = [0.15, 0.49] and OR = 0.22, 95% CI = [0.07, 0.63], respectively. The outcome was heterogeneous (I^2^ = 86.8%, *P* = 0.0005) therefore the random effect model was used; however, the heterogeneity could not be solved by leaving a study out of the analysis, Fig. 5.

## Discussion

### Summary of the results

To our knowledge, this is the first study to perform a comprehensive review and network meta-analyses on the effect of metoclopramide in reducing acute migraine attacks compared to other antimigraine drugs. We found that its efficacy in decreasing headache scores was significantly lower than only granisetron and significantly higher than placebo and sumatriptan, and its ability to completely relieve headache and decrease the need for rescue medication was significantly higher than only placebo and valproate in only the need for rescue medication. Also, the recurrence rates were similar between all antimigraine drugs and metoclopramide significantly decreased the incidence of nausea. Minimal side effects were reported with metoclopramide and the only reported extrapyramidal side effects were dystonia and akathisia.

Regarding headache outcomes, many systematic reviews and meta-analyses proved its ability in decreasing headache scores [7, 39, 40]. This was in line with our results; however, our review included only the randomized controlled trials that investigated the metoclopramide alone without any combinations, besides we investigated different doses of metoclopramide at different times of follow-up.

Most studies reported a dose of 10 mg of IV metoclopramide and significant improvement was achieved by this dose [15, 29, 34, 36]. On the other hand, other studies investigated the same dose and showed no significant results even with the placebo [19, 35]. While only Talabi et al. investigated the dose of 20 mg of IV metoclopramide and found that it significantly decreased the headache score compared to sumatriptan after 1 h of administration [30]. Salazar-Zúñiga et al. compared the dose of 10 mg of metoclopramide with sumatriptan and found significant results after only 15 min not after this even when reaching 1 h [29]. These results of Talabi et al. and Salazar-Zúñiga et al. could give us a clue of decreasing headache symptoms by increasing the dose of metoclopramide and both doses are needed to be directly compared in future research [29, 30]. Regarding the route of administration, the IV route showed significant results in improving headache change [15, 18, 34, 36]. IM route showed significant improvement in Jones et al. compared to placebo [27] while in Tfelt-Hansen et al., no significant improvements were observed for both IM or suppository routes [22]. However, it was difficult to determine the best route for the administration of metoclopramide whether IM or IV based on a small number of studies and both routes could have the same effect which is needed to be investigated in future research.

Metoclopramide’s action was kept with increasing the time from administration as observed by Amiri et al. after 4 h and Khazaei et al. after 24 h [28, 32]. Moreover, this decrease in headache score after 24 h was not significantly different to dexamethasone, chlorpromazine, and ketorolac [28]. This could prove the extended action of metoclopramide and explain its superiority over most antimigraine drugs – except for prochlorperazine and chlorpromazine – in decreasing the need for rescue medication as we found in the network meta-analysis.

Metoclopramide also exhibits its antimigraine action by reducing nausea and vomiting actions that accompany migraine attacks [41]. This supported our results of its efficacy in reducing nausea and vomiting compared to placebo, prochlorperazine, and ibuprofen [22, 27, 36]. Also, no any included found higher efficacy of other antimigraine drugs compared to metoclopramide, only Amiri et al. found no significant difference between granisetron and metoclopramide [32].

Regarding the adverse effects, some extrapyramidal side effects were reported with metoclopramide like dystonia and akathisia as found in only two of our included studies which were treated by diphenhydramine [15, 19]. However, these symptoms were also reported with prochlorperazine and bupivacaine [15, 37]. Moreover, tardive dyskinesia was also reported with metoclopramide and could be permanent. Also, some mild side effects were reported as dizziness, drowsiness, gastrointestinal symptoms, anxiety, dry mouth and blurred vision [18, 27–29, 33–35, 38]. The majority of included studies found that metoclopramide had less or the same rate of side effects compared with placebo or other drugs while it was associated with higher side effects compared to ketorolac and dexamethasone only [28, 33–35]. These findings could make us conclude that it had nearly the same rate of complications compared with other drugs. Also, other studies supported what we found [39, 42].

### Explanation of the results and mechanism of action

Metoclopramide exhibits its action through antagonize the dopaminergic receptor that has a role in migraine pathophysiology also enables metoclopramide to decrease nausea and vomiting action that accompany the migraine attacks [41, 43]. The ability to decrease pain can be also explained by its ability to decrease the central action of the trigeminovascular system [44]. This is performed through its ability to decrease the c-fos biomarker in the trigeminal nucleus caudalis [45]; however, its role as a c-fos biomarker is debatable in the literature [46, 47].

### The implication of the results

Our study presents a comprehensive review of metoclopramide use in migraine. Also, provide evidence about its superiority over other antimigraine drugs which can enable physicians to use it in acute emergencies of migraine by IV route mainly and with a dose of 10 mg. Twenty mg can be used in severe attacks. Moreover, its efficacy was significantly lower than granisetron in the network meta-analysis of headache change and significantly lower than prochlorperazine in the direct analysis of rescue medication. All of these findings make its use recommended and safe in relieving acute attacks.

### Strengths and limitations of the study

The main strength of our study was including a large number of studies with different routes and doses of metoclopramide in an attempt to conclude the best route and dose of metoclopramide. We also included studies that investigated metoclopramide alone without combination and we performed network meta-analyses to give a clue about the ranking of metoclopramide between other antimigraine drugs. However, we had some limitations like a limited number of studies to compare metoclopramide to other drugs to perform direct meta-analysis as the included studies were not sufficient to directly compare metoclopramide with other drugs. Also, network meta-analysis was limited as it included only studies that investigated metoclopramide, not all other studies that investigated other drugs which explained the inferiority of sumatriptan in the analyses despite it being one of the first-line treatments in relieving acute attacks. Moreover, the network meta-analyses showed high degrees of heterogeneity which could be explained by the small number of included studies in the analysis and the different subtypes of migraine whether episodic or chronic which was not specified in the included studies except in Salazar-Zúñiga et al. which reported that all patients had an episodic migraine while other studies did not report this information [29]. Therefore, we recommend further studies to specify the types of migraine and to perform a network meta-analysis comparing all antimigraine drugs in relieving acute migraine attacks.

## Conclusion

A dose of 10 mg of metoclopramide was effective in reducing acute migraine attacks, it only showed a lower significant effect compared with granisetron regarding headache change while it showed significantly higher effects only with placebo in both rescue medication needs and headache-free symptoms and valproate in only rescue medication need. Also, it significantly decreased headache scores more than placebo and sumatriptan. It had less or the same rates of side effects and recurrence of migraine attacks compared to other drugs. It was superior to other drugs to reduce nausea and vomiting except for granisetron which had the same effect. All of these findings may consider metoclopramide to be one of the first-line treatments to decrease acute migraine attacks in the emergency department.

## Supplementary Information

**Additional file 1: Supplementary Figure 1.** Headache change metoclopramide against placebo.

**Additional file 2: Supplementary Figure 2.** Headache change Metoclopramide against Chlorpromazine.

**Additional file 3: Supplementary Figure 3.** Headache complete relief metoclopramide against placebo.

**Additional file 4: Supplementary Figure 4.** Rescue Medication Metoclopramide against Placebo.

**Additional file 5: Supplementary Figure 5.** Rescue Medication Metoclopramide against Prochlorperazine.

**Additional file 6: Supplementary Table 1.** Headache change.

**Additional file 7: Supplementary Table 2.** Complete headache relief.

**Additional file 8: Supplementary Table 3.** Rescue medication need.

**Additional file 9: Supplementary Table 4.** Recurrence of attacks.

**Additional file 10: Supplementary Table 5.** Side effects.

**Additional file 11: Supplementary Table 6.** Nausea and emesis change.

**Additional file 12: Supplementary Table 7.** Nausea and emesis incidence.

## Data Availability

The datasets used and/or analyzed during the current study are not publicly available due to the difficulty of the organization of the data to be suitable for publication; however, they are available from the corresponding author upon reasonable request.

## Abbreviations

NMAs: Network Meta-Analyses
SMD: Standardized Mean Difference
CI: Confidence Interval
VAS: Visual Analogue Scale
NOS: Numeric Rating Scale
ORs: Odds Ratios
IV: Intravenous
IM: Intramuscular
SC: Subcutaneous
