# Supplementary figures and images for: The efficacy and safety of metoclopramide in relieving acute migraine attacks compared with other anti-migraine drugs: a systematic review and network meta-analysis of randomized controlled trials
Source: BMC Neurol. 2023 Jun 8;23:221. doi: 10.1186/s12883-023-03259-7 (PMC10249175; doi:10.1186/s12883-023-03259-7)

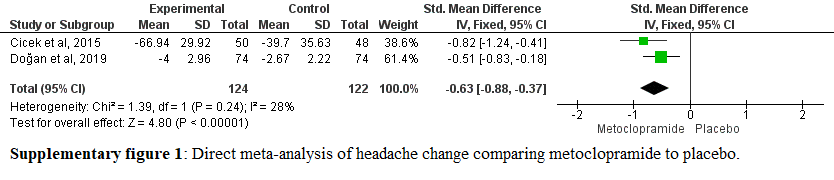

Supplement: Supplementary file 1 — Additional file 1: Supplementary Figure 1. Headache change metoclopramide against placebo. [file 12883_2023_3259_MOESM1_ESM.png]

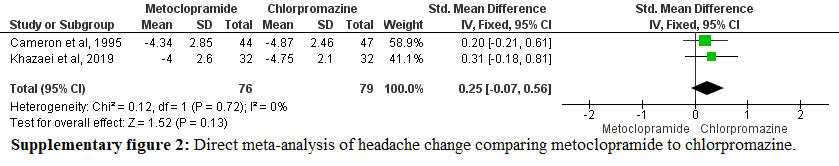

Supplement: Supplementary file 2 — Additional file 2: Supplementary Figure 2. Headache change Metoclopramide against Chlorpromazine. [file 12883_2023_3259_MOESM2_ESM.png]

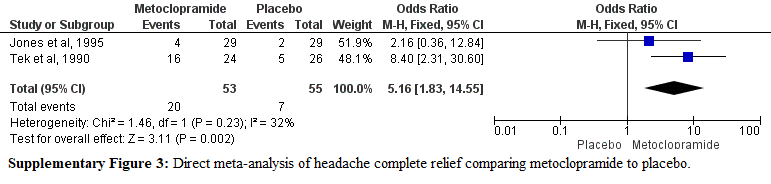

Supplement: Supplementary file 3 — Additional file 3: Supplementary Figure 3. Headache complete relief metoclopramide against placebo. [file 12883_2023_3259_MOESM3_ESM.png]

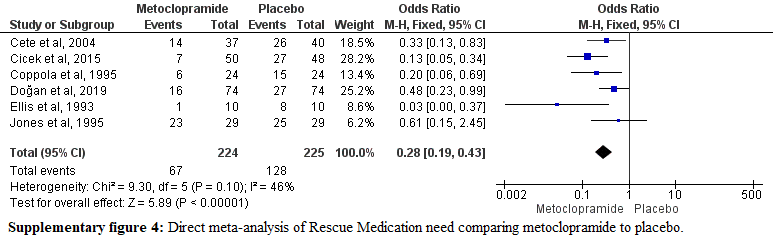

Supplement: Supplementary file 4 — Additional file 4: Supplementary Figure 4. Rescue Medication Metoclopramide against Placebo. [file 12883_2023_3259_MOESM4_ESM.png]

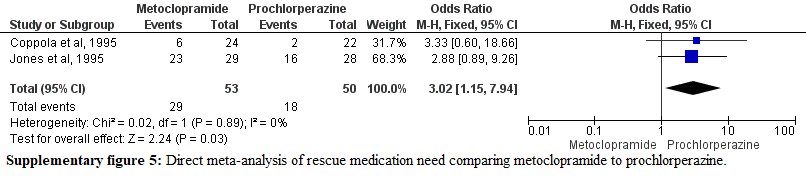

Supplement: Supplementary file 5 — Additional file 5: Supplementary Figure 5. Rescue Medication Metoclopramide against Prochlorperazine. [file 12883_2023_3259_MOESM5_ESM.png]
