# Supplementary material for: The efficacy and safety of metoclopramide in relieving acute migraine attacks compared with other anti-migraine drugs: a systematic review and network meta-analysis of randomized controlled trials
Source: BMC Neurol. 2023 Jun 8;23:221. doi: 10.1186/s12883-023-03259-7 (PMC10249175; doi:10.1186/s12883-023-03259-7)
Supplement: Supplementary file 6 — Additional file 6: Supplementary Table 1. Headache change. [file 12883_2023_3259_MOESM6_ESM.docx]

Supplementary Table 1: Headache change

| **Study ID** | **Drugs / Groups** | **Scale used** | **Results** | | | | | **P value, 95% CI** | | |
| --- | --- | --- | --- | --- | --- | --- | --- | --- | --- | --- |
| **Tfelt-Hansen et al, 1980** | | Rating scale of headache  0 = None  1 = Mild  2 = Moderate  3 = Severe  The excellent result for analgesic sedative ttt was the decrease of 2 points or reaching 0 on the rating scale with no further ttt and a stay in clinic less than 8 h. |  | | | | | | | |
|  |  |  |  |  |  |  |  |  |  |  |
|  |  |  | Baseline | 1 h (Excellent Results) | | 1 h (Unsatisfactory) | | P value | | |
|  | Metoclopramide 10 mg IM + Placebo suppository (49 pts) |  | All patients:  Severe: 71%  Moderate: 28%  Mild: 1% | 19 / 40 | | 21 / 40 | | P = 0.06 | | |
|  | Placebo IM + Placebo suppository (51 pts) |  |  | 18 / 47 | | 29 / 47 | |  |  |  |
|  | Metoclopramide 20 mg suppository + Placebo IM (50 pts) |  |  | 29 / 49 | | 20 / 49 | |  |  |  |
| **Tek et al, 1990** | | Numeric relief scores  4 = Completely relieved  3 = Mostly relieved  2 = Partially relieved  1 = No between at all  0 = Worse | Baseline | | 1 h |  | | P value | | |
|  | Metoclopramide 10 mg IV (24 pts) |  | -- | | Mean 2.46  SEM 0.2480 |  | | P < 0.02 | | |
|  | Normal saline 2 ml (26 pts) |  | -- | | Mean 1.69  SME 0.1903 |  | |  |  |  |
| **Ellis et al, 1993** | | VAS  10-cm horizontal visual analog scale | Metoclopramide was compared with each drug separately | | | | | | | |
|  |  |  | Baseline | 30 minutes | | 1 h | | After applying Bonferroni adjustment, metoclopramide was significantly better than placebo and ibuprofen  (adj-α = 0.0167) | | |
|  | Metoclopramide 10 mg IV + Placebo oral (10 pts) |  | Median 8.5 | Median 5 | | Median 1 | |  |  |  |
|  | Placebo oral and IV (10 pts) |  | Median 8  P: NS | Median 7  (P = 0.0449) | | Median 5.5  (P = 0.0013) | |  |  |  |
|  | Ibuprofen 600 mg oral + Placebo IV (10 pts) |  | Median 8.5  P: NS | Median 7  (P = 0.0443) | | Median 6  (P = 0.0135) | |  |  |  |
| **Cameron et al, 1995** | | VAS  10-cm visual analog scale | Baseline | 45 minutes | | Change | | P value | | |
|  | Metoclopramide 0.1 mg/kg IV (44 pts) |  | Mean 7.76 | Mean 3.46 | | Mean 4.34  SD 2.85 | | Mean difference 0.53 cm  P = 0.35  95% CI (0.001 to 1.62) | | |
|  | Chlorpromazine 0.1 mg/kg IV (47 pts) |  | Mean 7.15 | Mean 2.28 | | Mean 4.87  SD 2.46 | |  |  |  |
| **Jones et al, 1995** | | VAS  10-cm horizontal visual analog scale | Baseline | 1 h | | Change | | | P value | |
|  | Metoclopramide 10 mg IM (29 pts) |  | Median 8.5  Range (7-10) | Median 5.6 | | Median 2.9  Decreased severity of headache by 34% | | | P < 0.01 | |
|  | Normal saline 2 ml IM (29 pts) |  | Median 8  Range (6-10) | Median 6.7 | | Median 1.3  Decreased severity of headache by 16% | | |  |  |
|  | Prochlorperazine 10 mg IM (28 pts) |  | Median 8.1  Range (6-10) | Median 2.7 | | Median 5.4  Decreased severity of headache by 65% | | |  |  |
| **Coppola et al, 1995** | | VAS  10-centimeter non hatched visual analog scale | Baseline | | 30 minutes | | P value | | | |
|  | Metoclopramide 10 mg IV (24 pts) |  | Median 8.1 | | Median 3.9 | | P = 0.003 | | | |
|  | Normal saline 2 ml IV (24 pts) |  | Median 8.7 | | Median 1.1 | |  |  |  |  |
|  | Prochlorperazine 10 mg IV (22 pts) |  | Median 7.6 | | Median 6.1 | |  |  |  |  |
| **Cicek et al, 2004** | | VAS  100 mm visual analogue scale | Baseline | | 45 minutes | | P value | | | |
|  | Metoclopramide 10 mg IV + Placebo IM (50 pts) |  | Not reported | | Not reported | | Placebo was significantly higher than other gps (P = 0.000)  When exclude placebo, Pethidine was significantly higher than metoclopramide P = 0.04 | | | |
|  | Placebo IV + Placebo IM (48 pts) |  |  |  |  |  |  |  |  |  |
|  | Pethidine 50 mg IM + Placebo IV (49 pts) |  |  |  |  |  |  |  |  |  |
| **Cete et al, 2004** | | VAS  100 mm visual analogue scale | Baseline | 15 minutes | | 30 minutes |  | |  | |
|  | Metoclopramide 10 mg IV + 100 ml normal saline (37 pts) |  | Mean 73  SD 25 | Mean 51.73 | | Mean 33.19 | Changes of all gps compared with baseline at 30 minutes were significant in (P < 0.000)  The differences between groups weren't significant at 15, and 30 minutes (P = 0.619)  The differences between groups in patients of migraine with aura was significant at 15 minutes (P = 0.04), MgSO4 decreased pain significantly better than metoclopramide (P = 0.03) and placebo (P = 0.04) | | | |
|  | Normal saline 100 ml IV (40 pts) |  | Mean 69  SD 19 | Mean 52.88 | | Mean 44.19 |  |  |  |  |
|  | MgSO4 2 mg + 100 ml normal saline (36 pts) |  | Mean 70  SD 22 | Mean 45.97 | | Mean 35.6 |  |  |  |  |
| **Salazar-Zúñiga et al, 2006** | | Headache intensity scale;  0 = no headache  1 = mild  2 = moderate  3 = intense | Number of patients at each grade according to time | | | | | | P value | |
|  |  |  | Baseline | 15 minutes | 30 minutes | 45 minutes | 60 minutes | | The difference between gps was only significant at 15 minutes (P < 0.01) | |
|  | Metoclopramide 10 mg IV (60 pts) |  | 0 = 0  1 = 0  2 = 9  3 = 51 | 0 = 33  1 = 9  2 = 12  3 = 6 | 0 = 39  1 = 11  2 = 7  3 = 3 | 0 = 47  1 = 4  2 = 9  3 = 0 | 0 = 54  1 = 6  2 = 0  3 = 0 | |  |  |
|  | Sumatriptan 6 mg SC (60 pts) |  | 0 = 0  1 = 0  2 = 5  3 = 55 | 0 = 0  1 = 35  2 = 14  3 = 10 | 0 = 34  1 = 12  2 = 10  3 = 4 | 0 = 45  1 = 6  2 = 7  3 = 2 | 0 = 47  1 = 7  2 = 5  3 = 1 | |  |  |
| **Talabi et al, 2013** | | VAS  10-cm visual analog scale | Baseline | | 1 h | | P value | | | |
|  | Metoclopramide 20 mg IV (62 pts) |  | Mean 6.74  SD 0.84 | | Mean 0.66  SD 0.59 | | Mean difference 0.55 ± 0.13  P < 0.001  95% CI (0.25 – 0.79) cm | | | |
|  | Sumatriptan 6 mg SC (62 pts) |  | Mean 6.12  SD 0.73 | | Mean 1.1  SD 0.7 | |  |  |  |  |
| **Friedman et al, 2014** | | Verbal NRS  Patient assess pain between 0 (no pain) and 10 (worst imaginable)  &  Descriptive ordinal scale  Patient assess pain as  None - Mild - Moderate - Severe | Baseline | | 1 h | | P value | | | |
|  | Metoclopramide 10 mg IV (110 pts) |  | Median 9  IQR (8-10) | | Patients improved by 4.7 points  95% CI (4.2 - 5.2) | | When applying Bonferroni correction to account for the 3 overlapping pairwise comparisons:  Valproate VS Metoclopramide  Mean difference -1.9  95% CI (-2.8 , -1.1)  Valproate VS Ketorolac  Mean difference -1.1  95% CI (-2 , -0.2)  Metoclopramide VS Ketorolac  Mean difference 0.8  95% CI (-0.1 , 1.7) | | | |
|  | Ketorolac 30 mg IV (110 pts) |  | Median 8  IQR (7-10) | | Patients improved by 3.9 points  95% CI (3.3 - 4.5) | |  |  |  |  |
|  | Valproate 1 gm IV (110 pts) |  | Median 8  IQR (8-10) | | Patients improved by 2.8 points 95% CI (2.3 - 3.3) | |  |  |  |  |
| **Amiri et al, 2017** | | VAS | Baseline | 1h | 2h | 4h | P value | | | |
|  | Metoclopramide 10 mg IV (73 pts) |  | Mean 7.68  SD 1.13 | Mean 5.04  SD 1.77 | Mean 4.1  SD 1.8 | Mean 1.56  SD 0.68 | P = 0.03 | | | |
|  | Granisetrone 2 mg IV (75 pts) |  | Mean 7.67  SD 1.3 | Mean 3.2  SD 1.37 | Mean 2.39  SD 1.28 | Mean 1.31  SD 0.52 |  |  |  |  |
| **Doğan et al, 2019** | | NRS  11‐point numeric rating scale (NRS) score (0 = no pain, 10 = worst possible pain) | Values, differences and 95% CI at 15, and 30 minutes | | | | | | | |
|  |  |  | Baseline | | 15 minutes | | 30 minutes | | |  |
|  |  |  |  |  | Values | Change | Values | | Change |  |
|  | Metoclopramide 10 mg IV (74 pts) |  | Median 8  IQR (7-10) | | Median 6  IQR (4-7) | Median 2  IQR (1-3) | Median 3  IQR (1-5) | | Median 4  IQR (2-6) | Absolute risk reduction 47%  95% CI  (23.3 , 71.6) |
|  | Normal saline 100 ml (74 pts) |  | Median 8  IQR (7-9) | | Median 6  IQR (5-8) | Median 1  IQR (0-3) | Median 5  IQR (2-7) | | Median 3  IQR (1-4) |  |
|  |  |  | Differences of median 0  95% CI (-1.4 , 1.4) | | Differences of median -1  95% CI (−2.1 , 0.1) | | Differences of median -1  95% CI (−2.1 , 0.1) | | |  |
| **Khazaei et al, 2019** | | VAS  A scale of 0 and 10 | Baseline | 1 h | | 24 h | P value | | | |
|  | Metoclopramide 10 mg IV (32 pts) |  | Mean 8.42  SD 1.455  Range (5,10) | Mean 4.42  SD 2.157  Range (0,9) | | Mean 2.06  SD 3.434  Range (0,10) | The effect of each drug with baseline was significant P < 0.05  However, the differences between drugs at different intervals were:  At baseline: P = 0.458  At 1 h: P = 0.368  At 24 h: P = 0.386 | | | |
|  | Dexamethasone 8 mg IV (32 pts) |  | Mean 8.69  SD 1.491  Range (5,10) | Mean 4.72  SD 1.708  Range (2,10) | | Mean 0.81  SD 1.491  Range (0,5) |  |  |  |  |
|  | Chlorpromazine 25 mg IV (32 pts) |  | Mean 8.97  SD 1.204  Range (6,10) | Mean 4.22  SD 1.718  Range (1,7) | | Mean 1.91  SD 3.145  Range (0,10) |  |  |  |  |
|  | Ketorolac 30 mg IV (32 pts) |  | Mean 8.76  SD 1.393  Range (5,10) | Mean 3.76  SD 1.969  Range (0,8) | | Mean 1.03  SD 1.960  Range (0,7) |  |  |  |  |
| **Yavuz et al, 2020** | | VAS  100 mm straight line marked with ‘no pain’ on one end (0 mm) and ‘worst pain’ (100 mm) | Baseline | 15 minutes | | 30 minutes | | | P value | |
|  |  |  |  | Values | Change | Values | Change | |  |  |
|  | Metoclopramide 10 mg IV (50 pts) |  | Mean 79.02  SD 9.6  95% CI (76.3 , 81.8) | Mean 61.7  SD 19.1  95%CI (56.3 , 67.1) | Mean 17.3  SD 15.7  95%CI (12.9 , 21.8) | Mean 35.3  SD 21.4  95% CI (29.2 , 41.4) | Mean 43.7  SD 20.8  95%CI (37.8 , 49.6) | | Metoclopramide VS Dexketoprofen trometamol:  At 15 minutes:  P = 0.618  Mean difference – 3.8  95% CI (-13.4 , 5.8)  At 30 minutes:  P = 0.862  Mean difference 2.2  95% CI (-12.1 , 7.7) | |
|  | Dexketoprofen trometamol 50 mg IV (50 pts) |  | Mean 78.02  SD 11.6  95% CI (74.7 , 81.3) | Mean 56.9  SD 23.4  95%CI (50.3 , 63.6) | Mean 21.1  SD 20.5  95%CI (15.3 , 26.9) | Mean 32.1  SD 23.6  95% CI (25.4 , 38.8) | Mean 45.9  SD 22.4  95% CI (39.6 , 52.2) | |  |  |
| **Friedman et al, 2020** | | 11-point scale  0= no pain,  10= imaginable pain  &  Descriptive headache intensity scale none, mild, moderate, severe | Baseline | | 1 h | |  | | P value | |
|  | Metoclopramide 10 mg IV (48 pts) |  | Moderate 13 (27%)  Severe 35 (73%) | | Mean improvement 6.1  95% CI (5.2 , 6.9)  Percent improvement of pain score:  Mean 66%, SD 31% | |  | | Mean difference -1.1 95% CI (-2.3 , 0.1) | |
|  | Bupivacaine 0.5% (6 mL) (51 pts) |  | Moderate 13 (25%)  Severe 38 (75%) | | Mean improvement 5  95% CI (4.1 , 5.8)  Percent improvement of pain score:  Mean 54%, SD 30% | |  | |  |  |

Table 3 describes Headache change among articles

IV: Intravenous, IM: Intramuscular, SC: Subcutaneous, SD: Standard Deviation, CI: Confidence Interval, IQR: Inter Quartile Range, NS: Non-significant, h: hour, VAS: Visual Analogue Scale, NRS: Numeric Rating Scale, VS: versus, pts: patients, ttt: treatment, gps: groups.
