# Supplementary material for: The efficacy and safety of metoclopramide in relieving acute migraine attacks compared with other anti-migraine drugs: a systematic review and network meta-analysis of randomized controlled trials
Source: BMC Neurol. 2023 Jun 8;23:221. doi: 10.1186/s12883-023-03259-7 (PMC10249175; doi:10.1186/s12883-023-03259-7)
Supplement: Supplementary file 7 — Additional file 7: Supplementary Table 2. Complete headache relief. [file 12883_2023_3259_MOESM7_ESM.docx]

Supplementary Table 2 headache relief and success of treatment

| **Study ID** | **Drugs / Groups** | **Evaluation methods** | | **Results** | | | | **P value** |
| --- | --- | --- | --- | --- | --- | --- | --- | --- |
| **Tfelt-Hansen et al, 1980** | | The excellent result for analgesic sedative treatment was the decrease of 2 points or reaching 0 on the rating scale with no further treatment and a stay in clinic less than 8 h. | | 1 h (Excellent result) | |  | |  |
|  | Metoclopramide 10 mg IM + Placebo suppository (49 pts) |  |  | 19 / 40 | |  | | P = 0.06 |
|  | Placebo IM + Placebo suppository (51 pts) |  |  | 18 / 47 | |  |  |  |
|  | Metoclopramide 20 mg suppository + Placebo IM (50 pts) |  |  | 29 / 49 | |  |  |  |
| **Tek et al, 1990** | | Patients had sufficient relief to allow discharge from the ED without further treatment | | 1 h | |  | |  |
|  | Metoclopramide 10 mg IV (24 pts) |  |  | 16 / 28 | |  | | P < 0.001 |
|  | Normal saline 2 ml (26 pts) |  |  | 5 / 26 | |  |  |  |
| **Cameron et al, 1995** | | > 70% relief of pain on VAS | > 90% improvement | 100% improvement / pain free at discharge | | | | P value |
|  |  |  |  | After one dose | | 45 minutes | | **--** |
|  | Metoclopramide 0.1 mg/kg IV (44 pts) | 29 / 44 | 23 / 44 | 8 of 11who had complete relief at 45 minutes  Average doses of drugs given to pts 2.31 per patient | | 11 / 44 | |  |
|  | Chlorpromazine 0.1 mg/kg IV (47 pts) | 37 / 47 | 25 / 47 | 3 of 12 who had complete relief at 45 minutes  Average doses of drugs given to pts 2.38 per patient | | 12 / 47 | |  |
| **Jones et al, 1995** | | Headache relief at 1 h  However, the 13 pts of metoclopramide, and prochlorperazine had a complete relief within an average of 38 minutes, and range (28 to 60 minutes) | | | | | | |
|  |  |  |  |  |  |  |  |  |
|  |  | Headache worse | No relief | Partial relief | | Complete relief | | P value |
|  | Metoclopramide 10 mg IM (29 pts) | 3 / 29 | 12 / 29 | 10 / 29 | | 4 / 29 | | P = 0.04 |
|  | Normal saline 2 ml IM (29 pts) | 5 / 29 | 14 / 29 | 8 / 29 | | 2 / 29 | |  |
|  | Prochlorperazine 10 mg IM (28 pts) | 2 / 28 | 6 / 28 | 11 / 28 | | 9 / 28 | |  |
| **Coppola et al, 1995** | | Successful treatment:  Patient satisfaction and either a decrease of 50% or more in the 30-minute pain score (compared with the initial score) or an absolute pain score of 2.5 cm or less | | 30 minutes | |  | |  |
|  | Metoclopramide 10 mg IV (24 pts) |  |  | 48% | | Metoclopramide VS Placebo  P = 0.37  Prochlorperazine VS Metoclopramide  P = 0.03  Prochlorperazine VS Placebo  P = 0.001 | | |
|  | Normal saline 2 ml IV (24 pts) |  |  | 29% | |  |  |  |
|  | Prochlorperazine 10 mg IV (22 pts) |  |  | 82% | |  |  |  |
| **Cicek et al, 2004** | | Success treatment | | Success treatment at 45 minutes | |  | P value | |
|  | Metoclopramide 10 mg IV + Placebo IM (50 pts) |  |  | 86% | |  | **--** | |
|  | Placebo IV + Placebo IM (48 pts) |  |  | 43% | |  |  |  |
|  | Pethidine 50 mg IM + Placebo IV (49 pts) |  |  | **--** | |  |  |  |
| **Salazar-Zúñiga et al, 2006** | | Headache intensity scale;  0 = no headache  1 = mild  2 = moderate  3 = intense | Having no headache at different time intervals | | | | P value | |
|  |  |  | 15 minutes | 30 minutes | 45 minutes | 1 h | The difference between groups was only significant at 15 minutes (P < 0.01) | |
|  | Metoclopramide 10 mg IV (60 pts) |  | 33 / 60 | 39 / 60 | 47 / 60 | 54 / 60 |  |  |
|  | Sumatriptan 6 mg SC (60 pts) |  | 0 / 60 | 34 / 60 | 45 / 60 | 47 / 60 |  |  |
| **Friedman et al, 2014** | | Descriptive ordinal scale  Patient assess pain as  none, mild, moderate, severe | 2 h headache freedom;  Achieved level "none" within 2 h | | Sustained headache freedom;  Achieved level "none" within 2 h and continue till 24 h | | P value | |
|  |  |  |  |  |  |  |  |  |
|  | Metoclopramide 10 mg IV (110 pts) |  | 31 / 110 , 95% CI (21 , 37) | | 12 / 109 , 95% CI (6 , 18) | | **--** | |
|  | Ketorolac 30 mg IV (110 pts) |  | 27 / 110 , 95% CI (17 , 33) | | 17 / 109 , 95% CI (10 , 24) | |  |  |
|  | Valproate 1 gm IV (110 pts) |  | 16 / 110, 95% CI (9 , 22) | | 4 / 110 , 95% CI (1 , 9) | |  |  |
| **Doğan et al, 2019** | | No pain after discharge, it was assessed 24 – 72 h after discharge using a telephone survey | | 24 – 72 h | |  |  | |
|  | Metoclopramide 10 mg IV (74 pts) |  |  | 40 / 74 | |  | Difference 6.4%  95% CI(−33.0 , 46.0) | |
|  | Normal saline 100 ml (74 pts) |  |  | 33 / 74 | |  |  |  |
| **Friedman et al, 2020** | | Descriptive headache intensity scale;  none, mild, moderate, severe | Sustained headache relief;  Achieved level of "mild/none" within 2h in ED, not need additional analgesics in ED, or home, and not relapse to a level worse than mild for the next 48 after drug administration | | | Sustained headache freedom;  Achieved level of "none" in 2 h, and maintain it for 48 h without use additional analgesics | | |
|  |  |  |  |  |  |  |  |  |
|  | Metoclopramide 10 mg IV (48 pts) |  | Yes 18 / 48 , No 29 / 48 , Missing 1 | | | Yes 7 / 48 , No 41 / 48 | | |
|  | Bupivacaine 0.5% (6 mL) (51 pts) |  | Yes 11 / 51 , No 40 /51 , Missing 0 | | | Yes 3 / 51 , No 48 / 51 | | |

Table 4 describes number of patients who had headache relief and success of treatment through different time intervals among articles

IV: Intravenous, IM: Intramuscular, CI: Confidence Interval, h: hour, VAS: Visual Analogue Scale, VS: versus, ED: emergency department, pts: patients.
