# Supplementary material for: The efficacy and safety of metoclopramide in relieving acute migraine attacks compared with other anti-migraine drugs: a systematic review and network meta-analysis of randomized controlled trials
Source: BMC Neurol. 2023 Jun 8;23:221. doi: 10.1186/s12883-023-03259-7 (PMC10249175; doi:10.1186/s12883-023-03259-7)
Supplement: Supplementary file 8 — Additional file 8: Supplementary Table 3. Rescue medication need. [file 12883_2023_3259_MOESM8_ESM.docx]

Supplementary Table 3 rescue medication need

| **Study ID** | **Drugs / Groups** | **Name of drug** | **Results** | | **P value, 95% CI** |
| --- | --- | --- | --- | --- | --- |
| **Ellis et al, 1993** | | If patient hadn't achieved complete relief of symptoms after 1 h, they were given additional medication according to physician's choice 1 h | | | P value |
|  |  |  |  |  |  |
|  | Metoclopramide 10 mg IV + Placebo oral (10 pts) | 1 / 10 | | | **--** |
|  | Placebo oral and IV (10 pts) | 8 / 10  1 received 10 mg metoclopramide, one noted complete relief and the other noted feeling better at discharge | | |  |
|  | Ibuprofen 600 mg oral + Placebo IV (10 pts) | 7 / 10  1 received 10 mg metoclopramide, and noted significant improvement | | |  |
| **Cameron et al, 1995** | | Drug name: The other study drug or meperidine with dimenhydrinate.  Early treatment failure, when patient required additional medication after taking 3 doses of study drug by 1 h while being in ED | | | P value |
|  |  |  |  |  |  |
|  | Metoclopramide 0.1 mg/kg IV (44 pts) | 11 / 44 , 25% failure rate | | | P = 0.5 |
|  | Chlorpromazine 0.1 mg/kg IV (47 pts) | 9 / 47 , 19.1% failure rate | | |  |
| **Jones et al, 1995** | | Parenteral narcotics (ie, meperidine, morphine, or hydromorphone) | 1 h | | P value |
|  | Metoclopramide 10 mg IM (29 pts) |  | 23 / 29 | | P = 0.03 |
|  | Normal saline 2 ml IM (29 pts) |  | 25 / 29 | |  |
|  | Prochlorperazine 10 mg IM (28 pts) |  | 16 / 28 | |  |
| **Coppola et al, 1995** | | It was administered by the choice of the treating physician 30 minutes |  | | P value |
|  |  |  |  |  |  |
|  | Metoclopramide 10 mg IV (24 pts) | 6 / 24  Three received 2 mg butorphanol and three received prochlorperazine, and all had complete relief of symptoms after additional 30 minutes | | | **--** |
|  | Normal saline 2 ml IV (24 pts) | 15 / 24  Eight received prochlorperazine, and only 1 of those required additional medication (butorphanol)  Five received metoclopramide, and 3 of those required additional analgesia (2 received butorphanol and 1 received meperidine)  One received dihydroergotamine and One received butorphanol. | | |  |
|  | Prochlorperazine 10 mg IV (22 pts) | 2 / 22  One was given metoclopramide with no improvement, the other was given 2 mg butorphanol with complete relief of symptoms after additional 30 minutes | | |  |
| **Cicek et al, 2004** | | If patient had poor pain relief, It was administered by the choice of the treating physician  The following drugs were used: metoclopramide, NSAIDs, and opioids | 1 h | | P value |
|  | Metoclopramide 10 mg IV + Placebo IM (50 pts) |  | 7 / 50 | | Patients with low vascular score had high rescue of analgesics  P = 0.042  Metoclopramide VS Placebo  P = 0.007  Metoclopramide VS Pethidine  P = 0.000 |
|  | Placebo IV + Placebo IM (48 pts) |  | 27 / 48 | |  |
|  | Pethidine 50 mg IM + Placebo IV (49 pts) |  | 20 / 49 | |  |
| **Cete et al, 2004** | | 0.75 mg/kg meperidine  A repeat dose of meperidine was administered 30 min later if needed | 30 minutes | | P value |
|  | Metoclopramide 10 mg IV + 100 ml normal saline (37 pts) |  | 14 / 37 (38%) | | Placebo was significantly higher than other drugs  P = 0.04 |
|  | Normal saline 100 ml IV (40 pts) |  | 26 / 40 (65%) | |  |
|  | MgSO4 2 mg + 100 ml normal saline (36 pts) |  | 16 / 36 (44%) | |  |
| **Friedman et al, 2014** | | It was determined by the treating physician | 1 h | | P value |
|  | Metoclopramide 10 mg IV (110 pts) |  | 36 / 110 , 95% CI (25 , 42) | | **--** |
|  | Ketorolac 30 mg IV (110 pts) |  | 57 / 110 , 95% CI (43 , 61) | |  |
|  | Valproate 1 gm IV (110 pts) |  | 76 / 110 , 95% CI (60 , 77) | |  |
| **Doğan et al, 2019** | | For patients who expressed refractory headache at 30 minutes they were given 1 μg/kg of fentanyl | 30 minutes | |  |
|  | Metoclopramide 10 mg IV (74 pts) |  | 16 / 74 (21.6%) | | Mean difference −14.9%  95% CI (−32.0 , 2.5) |
|  | Normal saline 100 ml (74 pts) |  | 27 / 74 (36.5%) | |  |
| **Yavuz et al, 2020** | | If any patient had same pain intensity at the end of 30 minutes and those who required additional drug they were given IV infusion of 1 μg/kg fentanyl | 30 minutes | | P value |
|  | Metoclopramide 10 mg IV (50 pts) |  | 3 / 50 | | **--** |
|  | Dexketoprofen trometamol 50 mg IV (50 pts) |  | 4 / 50 | |  |
| **Friedman et al, 2020** | | **--** | 1 h | |  |
|  | Metoclopramide 10 mg IV (48 pts) |  | 8 / 48 (17%) | | Between group difference 17%  95% CI (0 , 33%) |
|  | Bupivacaine 0.5% (6 mL) (51 pts) |  | 17 / 51 (33%) | |  |

Table 5 describes the number of patients who needed additional analgesics rather than the study treatment

IV: Intravenous, IM: Intramuscular, CI: Confidence Interval, h: hour, VS: versus, ED: emergency department, NSAIDs: Non-steroidal anti-inflammatory drugs, pts: patients.
