# Supplementary material for: The efficacy and safety of metoclopramide in relieving acute migraine attacks compared with other anti-migraine drugs: a systematic review and network meta-analysis of randomized controlled trials
Source: BMC Neurol. 2023 Jun 8;23:221. doi: 10.1186/s12883-023-03259-7 (PMC10249175; doi:10.1186/s12883-023-03259-7)
Supplement: Supplementary file 9 — Additional file 9: Supplementary Table 4. Recurrence of attacks. [file 12883_2023_3259_MOESM9_ESM.docx]

Supplementary Table 4. recurrence of attacks

| **Study ID** | **Drugs / Groups** | **Results** | | | **P value** |
| --- | --- | --- | --- | --- | --- |
| **Tek et al, 1990** | | Within 48 h there was no return of patients to ED due to recurrence; however, the authors weren't sure if they went to another ED due to recurrence or not | | | |
|  | Metoclopramide 10 mg IV (24 pts) |  |  |  |  |
|  | Normal saline 2 ml (26 pts) |  |  |  |  |
| **Cameron et al, 1995** | | Patients with early treatment failure (11 in metoclopramide, and 9 in chlorpromazine) were excluded from counting the recurrence of pain which was defined as late treatment failure. Also, late treatment failure was detected by nurse during a telephone follow-up after 48 h | | |  |
|  |  |  |  |  |  |
|  | Metoclopramide 0.1 mg/kg IV (44 pts) | They contacted only 29 of 33 to check for recurrence  There was 16 / 29 who reported recurrence  Of the 16 there was:  10 had mild recurrence, 8 of them took medications, but only 4 need drugs stronger than acetaminophen or aspirin (e.g. codeine)  6 had severe recurrence, 5 of them returned to ED, and one visited the family physician | | | The difference between the 2 groups for both and mild, moderate recurrence were non-significant.  For mild, P = 0.17  For severe, P = 0.28 |
|  | Chlorpromazine 0.1 mg/kg IV (47 pts) | They contacted only 35 of 38 to check for recurrence  There was 21 / 35 who reported recurrence  Of the 21 there was:  18 had mild recurrence, 14 of them took medications, but only 6 need drugs stronger than acetaminophen or aspirin  3 had severe recurrence, 1 returned to ED, and 1 visited the family physician | | |  |
| **Coppola et al, 1995** | |  | 24 h | 48 h | P value |
|  | Metoclopramide 10 mg IV (24 pts) |  | 1 / 24 | They had contacted only 22 patients (31%), and none of them had relapse of migraine | **--** |
|  | Normal saline 2 ml IV (24 pts) |  | 3 / 24 |  |  |
|  | Prochlorperazine 10 mg IV (22 pts) |  | 0 / 22 |  |  |
| **Cete et al, 2004** | |  | 24 h |  | P value |
|  | Metoclopramide 10 mg IV + 100 ml normal saline (37 pts) |  | 16 / 37 (43%) |  | P > 0.05 |
|  | Normal saline 100 ml IV (40 pts) |  | 21 / 40 (52%) |  |  |
|  | MgSO4 2 mg + 100 ml normal saline (36 pts) |  | 19 / 36 (52%) |  |  |
| **Doğan et al, 2019** | |  | Patients who revisited ED within 24 – 72 h after discharge | |  |
|  | Metoclopramide 10 mg IV (74 pts) |  | 3 / 74 (6.7%) |  | Difference **-**3.3%  95% CI (-15.6 , 8.9) |
|  | Normal saline 100 ml (74 pts) |  | 4 / 74 (10%) |  |  |
| **Khazaei et al, 2019** | |  | 6 / 32 |  | P value |
|  | Metoclopramide 10 mg IV (32 pts) |  |  |  | P = 0.05 |
|  | Dexamethasone 8 mg IV (32 pts) |  | 0 / 32 |  |  |
|  | Chlorpromazine 25 mg IV (32 pts) |  | 5 / 32 |  |  |
|  | Ketorolac 30 mg IV (32 pts) |  | 4 / 32 |  |  |

Table 6 describes the recurrence of the migraine attacks among participants

IV: Intravenous, IM: Intramuscular, CI: Confidence Interval, h: hour, VS: versus, ED: emergency department, pts: patients.
