# Supplementary material for: The efficacy and safety of metoclopramide in relieving acute migraine attacks compared with other anti-migraine drugs: a systematic review and network meta-analysis of randomized controlled trials
Source: BMC Neurol. 2023 Jun 8;23:221. doi: 10.1186/s12883-023-03259-7 (PMC10249175; doi:10.1186/s12883-023-03259-7)
Supplement: Supplementary file 10 — Additional file 10: Supplementary Table 5. Side effects. [file 12883_2023_3259_MOESM10_ESM.docx]

Supplementary Table 5, side effects

| **Study ID** | **Drugs / Groups** | **Side effect** | | | **results** | |  | | **P value** | |
| --- | --- | --- | --- | --- | --- | --- | --- | --- | --- | --- |
| **Tfelt-Hansen et al, 1980** | | No side effects noted | | | | | | | | |
|  | Metoclopramide 10 mg IM + Placebo suppository (49 pts) |  |  |  |  |  |  |  |  |  |
|  | Placebo IM + Placebo suppository (51 pts) |  |  |  |  |  |  |  |  |  |
|  | Metoclopramide 20 mg suppository + Placebo IM (50 pts) |  |  |  |  |  |  |  |  |  |
| **Tek et al, 1990** | | No dystonic reactions, hypotension, sedation, or other serious or clinically significant side effects | | | | | | |  | |
|  |  |  |  |  |  |  |  |  |  |  |
|  |  |  | | | 1 h | |  | | P value | |
|  | Metoclopramide 10 mg IV (24 pts) | Restlessness | | | 2 / 24 | |  | | **--** | |
|  | Normal saline 2 ml (26 pts) |  |  |  | 1 / 26 | |  | |  |  |
| **Cameron et al, 1995** | | Drowsiness, Dizziness, Nasal Congestion, Dry mouth, Nausea, Vomiting, Dyspepsia, Nervousness, Tremulousness, and significant drop in BP | | | 45 minutes | | | | P value | |
|  | Metoclopramide 0.1 mg/kg IV (44 pts) |  |  |  | 13 / 29 | |  | | P = 0.94 | |
|  | Chlorpromazine 0.1 mg/kg IV (47 pts) |  |  |  | 16 / 35 | |  | |  |  |
| **Jones et al, 1995** | | There were no episodes of orthostatic hypotension, seizures, akathisia, or dystonic reactions encountered | | | | | | |  | |
|  |  |  |  |  |  |  |  |  |  |  |
|  |  |  | | | 1 h | |  | | P value | |
|  | Metoclopramide 10 mg IM (29 pts) | Drowsiness | | | 5 / 29 | |  | | **--** | |
|  | Normal saline 2 ml IM (29 pts) |  |  |  | 0 | |  | |  |  |
|  | Prochlorperazine 10 mg IM (28 pts) |  |  |  | 5 / 28 | |  | |  |  |
| **Coppola et al, 1995** | | Extrapyramid.al reactions in form of Dystonia or Akathisia. However, the authors used IV diphenhydramine for ablation these reactions | | | Within minutes of drug administration | | | | **--** | |
|  |  |  |  |  |  |  |  |  |  |  |
|  | Metoclopramide 10 mg IV (24 pts) |  |  |  | 2 / 24 | |  | |  |  |
|  | Normal saline 2 ml IV (24 pts) |  |  |  | 0 | |  | |  |  |
|  | Prochlorperazine 10 mg IV (22 pts) |  |  |  | 2 / 22 | |  | |  |  |
| **Cicek et al, 2004** | | Drowsiness / light sedation, Dizziness, Nausea, Dysphoria, Restlessness, and Flushing | | | For the 4 h after treatment | | | | P value | |
|  | Metoclopramide 10 mg IV + Placebo IM (50 pts) |  |  |  | 38% | |  | | P = 0.000  Placebo VS Metoclopramide  P = 0.001  Placebo VS Pethidine  P = 0.000 | |
|  | Placebo IV + Placebo IM (48 pts) |  |  |  | 12.5% | |  | |  |  |
|  | Pethidine 50 mg IM + Placebo IV (49 pts) |  |  |  | 57.1% | |  | |  |  |
| **Cete et al, 2004** | | None of the side-effects required discontinuation of the drug infusion. | | | | | | | **--** | |
|  |  |  |  |  |  |  |  |  |  |  |
|  | Metoclopramide 10 mg IV + 100 ml normal saline (37 pts) | Dystonic reaction after infusion, treated by diphenhydramine | | | 1 / 37 (3%) | |  | |  |  |
|  | Normal saline 100 ml IV (40 pts) | No side effects reported | | |  | |  | |  |  |
|  | MgSO4 2 mg + 100 ml normal saline (36 pts) | Flushing, it didn't require treatment | | | 3 / 36 (8%) | |  | |  |  |
| **Salazar-Zúñiga et al, 2006** | |  | | |  | |  | | **--** | |
|  | Metoclopramide 10 mg IV (60 pts) | The most frequently reported side effects were dizziness and drowsiness; however, they didn't last >20 minutes | | | | | | |  |  |
|  | Sumatriptan 6 mg SC (60 pts) | The most frequently one was momentary increase of the headache, mainly in the frontal region, then shortness of breath, pressure sensation, anxiety, fatigue, pain at the injection site, sweating, sensation of heat, and paresthesia of variable duration; however, they didn't last >40 minutes | | | | | | |  |  |
| **Talabi et al, 2013** | | No serious side effects e.g. dystonia, chest pain, or chest tightness were observed in both groups | | | | | | | | |
|  | Metoclopramide 20 mg IV (62 pts) |  |  |  |  |  |  |  |  |  |
|  | Sumatriptan 6 mg SC (62 pts) |  |  |  |  |  |  |  |  |  |
| **Friedman et al, 2014** | | 24 h; Dizziness, Upper gastrointestinal complaint (dyspepsia, heartburn, and bloating), Restlessness | | 1 h; drowsiness, it was measured using a degree of (none/ little drowsy but able to function/ too drowsy to function | | | | | | |
|  |  |  |  |  |  |  |  |  |  |  |
|  |  |  |  | None | | | | little drowsy but able to function | | too drowsy to function |
|  | Metoclopramide 10 mg IV (110 pts) | 24 / 109 , 95% CI (15 , 31) | | 67 / 109 , 95% CI (53 , 71) | | | | 39 | | 2 |
|  | Ketorolac 30 mg IV (110 pts) | 33 / 110 , 95% CI (22 , 39) | | 66 / 110 , 95% CI (51 , 69) | | | | 41 | | 2 |
|  | Valproate 1 gm IV (110 pts) | 25 / 110 , 95% CI (16 , 31) | | 69 / 110 , 95% CI (53 , 71) | | | | 34 | | 7 |
| **Doğan et al, 2019** | | No akathisia or dystonic reactions  They only developed dizziness | | | 30 minutes | | | |  | |
|  | Metoclopramide 10 mg IV (74 pts) |  |  |  | 4 / 74 (5.4%) | |  | | Difference %: 0%  95% CI (∞ , ∞) | |
|  | Normal saline 100 ml (74 pts) |  |  |  | 4 / 74 (5.4%) | |  | |  |  |
| **Khazaei et al, 2019** | |  | They were assessed at both intervals 1 h, 24 h | | | | | | P value | |
|  | Metoclopramide 10 mg IV (32 pts) | Dizziness, nausea, malaise, and insomnia | | | | 10 / 32 |  | | P < 0.0001 | |
|  | Dexamethasone 8 mg IV (32 pts) | Mood changes and insomnia | | | | 6 / 32 |  | |  |  |
|  | Chlorpromazine 25 mg IV (32 pts) | Dizziness, drowsiness, anxiety, dry mouth, blurred vision, and constipation | | | | 22 / 32 |  | |  |  |
|  | Ketorolac 30 mg IV (32 pts) | Upset stomach, dizziness, nausea, drowsiness | | | | 7 / 32 |  | |  |  |
| **Yavuz et al, 2020** | | No side effects observed in all groups | | | | | | | | |
|  | Metoclopramide 10 mg IV (50 pts) |  |  |  |  |  |  |  |  |  |
|  | Dexketoprofen trometamol 50 mg IV (50 pts) |  |  |  |  |  |  |  |  |  |
| **Friedman et al, 2020** | | Akathisia was assessed 30 minutes after drug administration.  It was assessed by asking patients to rate their anxiety and restlessness on 0 – 10 scale.  Bupivacaine was more protective than Metoclopramide regarding anxiety: (OR 0.35 , 95%CI 0.15 , 0.85)  However, it wasn't against restlessness: (OR 0.64 , 95% CI 0.29, 1.42) | | | | | | | | |
|  |  |  |  |  |  |  |  |  |  |  |
|  | Metoclopramide 10 mg IV (48 pts) | Dizziness, drowsiness, worsening or changed headache, injection site reaction | | | 18 / 48 (38%) | |  | | Difference % : 6%  95% CI (-13 , 25%) | |
|  | Bupivacaine 0.5% (6 mL) (51 pts) | Dizziness, worsening or changed headache, head numbness, injection site reaction, gastrointestinal symptoms | | | 16 / 51 (31%) | |  | |  |  |

Table 7 describes different side effects reported by patients with different study drugs

IV: Intravenous, IM: Intramuscular, SC: Subcutaneous, CI: Confidence Interval, IQR: Inter Quartile Range, VS: versus, OR: Odds ratio, pts: patients.
