# Supplementary material for: The efficacy and safety of metoclopramide in relieving acute migraine attacks compared with other anti-migraine drugs: a systematic review and network meta-analysis of randomized controlled trials
Source: BMC Neurol. 2023 Jun 8;23:221. doi: 10.1186/s12883-023-03259-7 (PMC10249175; doi:10.1186/s12883-023-03259-7)
Supplement: Supplementary file 11 — Additional file 11: Supplementary Table 6. Nausea and emesis change. [file 12883_2023_3259_MOESM11_ESM.docx]

Supplementary Table 6, nausea / emesis change

| **Study ID** | **Drugs / Groups** | **Scale used** | **Results** | | | | | | **P value** |
| --- | --- | --- | --- | --- | --- | --- | --- | --- | --- |
| **Tfelt-Hansen et al, 1980** | | Rating scale of nausea  0 = None  1 = Mild  2 = Moderate  3 = Severe  The excellent result for nausea ttt was the decrease of 2 points or reaching 0 on the rating scale within 1 h | Baseline | | 1 h  (Excellent Results) | | 1 h (Unsatisfactory) | |  |
|  |  |  |  |  |  |  |  |  |  |
|  | Metoclopramide 10 mg IM + Placebo suppository (49 pts) |  | All patients:  Severe: 63%  Moderate: 29%  Mild: 8% | | 37 | | 5 | | P = 0.04 |
|  | Placebo IM + Placebo suppository (51 pts) |  |  |  | 35 | | 14 | |  |
|  | Metoclopramide 20 mg suppository + Placebo IM (50 pts) |  |  |  | 41 | | 8 | |  |
| **Ellis et al, 1993** | | Nausea was assessed by VAS  10-cm horizontal visual analog scale | Baseline | | 30 minutes | | 1 h | |  |
|  | Metoclopramide 10 mg IV + Placebo oral (10 pts) |  | Median 4 | | Median 0 | | Median 0 | | **--** |
|  | Placebo oral and IV (10 pts) |  | Median 2.5 | | Median 0.5 | | Median 1.5 | |  |
|  | Ibuprofen 600 mg oral + Placebo IV (10 pts) |  | Median 4.5  P: NS | | Median 1.5  P: NS | | Median 0  P = 0.0433 | |  |
| **Coppola et al, 1995** | | Nausea was assessed by VAS  10-centimeter non hatched visual analog scale | Baseline | | 30 minutes | |  | |  |
|  | Metoclopramide 10 mg IV (24 pts) |  | Median 4.1 | | Median 2.4 | |  | | P = 0.15 |
|  | Normal saline 2 ml IV (24 pts) |  | Median 2.4 | | Median 1.2 | |  | |  |
|  | Prochlorperazine 10 mg IV (22 pts) |  | Median 3.9 | | Median 0.5 | |  | |  |
| **Amiri et al, 2017** | | Emesis was assessed by VAS | Baseline | 1 h | | 2 h | | 4 h |  |
|  | Metoclopramide 10 mg IV (73 pts) |  | Mean 1.8  SD 0.77 | Mean 1.38  SD 0.73 | | Mean 0.21  SD 0.47 | | Mean 0.41  SD 0.19 | P = 0.7 |
|  | Granisetrone 2 mg IV (75 pts) |  | Mean 1.85  SD 0.81 | Mean 1.33  SD 0.66 | | Mean 0.25  SD 0.49 | | Mean 0.04  SD 0.19 |  |

Table 8 describes the changes of nausea and emesis scores among patients

IV: Intravenous, IM: Intramuscular, SD: Standard Deviation, NS: Non-significant, h: hour, VAS: Visual Analogue Scale, pts: patients.
