# Supplementary material for: The efficacy and safety of metoclopramide in relieving acute migraine attacks compared with other anti-migraine drugs: a systematic review and network meta-analysis of randomized controlled trials
Source: BMC Neurol. 2023 Jun 8;23:221. doi: 10.1186/s12883-023-03259-7 (PMC10249175; doi:10.1186/s12883-023-03259-7)
Supplement: Supplementary file 12 — Additional file 12: Supplementary Table 7. Nausea and emesis incidence. [file 12883_2023_3259_MOESM12_ESM.docx]

Supplementary Table 7, nausea / emesis incidence

| **Study ID** | **Drugs / Groups** | **Symptoms** | **Results** | | **P value** |
| --- | --- | --- | --- | --- | --- |
| **Cameron et al, 1995** | | Nausea, vomiting, dyspepsia | 45 minutes |  | **--** |
|  | Metoclopramide 0.1 mg/kg IV (44 pts) |  | 1 / 35 |  |  |
|  | Chlorpromazine 0.1 mg/kg IV (47 pts) |  | 1 / 29 |  |  |
| **Jones et al, 1995** | | Nausea, and vomiting | Baseline | 1 h | P < 0.001 |
|  | Metoclopramide 10 mg IM (29 pts) |  | 21 / 29 | 11 / 21 had relieved nausea |  |
|  | Normal saline 2 ml IM (29 pts) |  | 24 / 29 | 3 / 24 had relieved nausea |  |
|  | Prochlorperazine 10 mg IM (28 pts) |  | 19 / 28 | 14 / 19 had complete relief of nausea |  |
| **Cicek et al, 2004** | | Nausea; they reported total number of patients together; (vascular headache, and tension headache) | For the 4 h after treatment | | **--** |
|  | Metoclopramide 10 mg IV + Placebo IM (50 pts) |  | 6 / 85 |  |  |
|  | Placebo IV + Placebo IM (48 pts) |  | 5 / 83 |  |  |
|  | Pethidine 50 mg IM + Placebo IV (49 pts) |  | 19 / 84 |  |  |

Table 9 describes the incidence of nausea, and emesis among patients

IV: Intravenous, IM: Intramuscular, h: hour, pts: patients.
